# Restoration of HBV-specific CD8^+^ T-cell responses by sequential low-dose IL-2 treatment in non-responder patients after IFN-α therapy

**DOI:** 10.1038/s41392-021-00776-0

**Published:** 2021-11-05

**Authors:** Dongyao Wang, Binqing Fu, Xiaokun Shen, Chuang Guo, Yanyan Liu, Junfei Zhang, Rui Sun, Ying Ye, Jiabin Li, Zhigang Tian, Haiming Wei

**Affiliations:** 1 Institute of Immunology and the CAS Key Laboratory of Innate Immunity and Chronic Disease, School of Basic Medicine and Medical Center, University of Science and Technology of China, Hefei, Anhui 230001 China; 2 Hefei National Laboratory for Physical Sciences at Microscale, University of Science and Technology of China, Hefei, Anhui 230001 China; 3 Department of Infectious Diseases, the First Affiliated Hospital of Anhui Medical University, Hefei, Anhui 230027 China

**Keywords:** Infectious diseases, Cell biology

## Abstract

Patients with chronic hepatitis B (CHB) undergoing interferon (IFN)-α-based therapies often exhibit a poor HBeAg serological response. Thus, there is an unmet need for new therapies aimed at CHB. This study comprised two clinical trials, including 130 CHB patients, who were treatment-naïve; in the first, 92 patients were systematically analyzed ex vivo for interleukin-2 receptor (IL-2R) expression and inhibitory molecules expression after receiving Peg-IFN-α-2b therapy. In our second clinical trial, 38 non-responder patients, in whom IFN-α therapy had failed, were treated with or without low-dose IL-2 for 24 weeks. We then examined the hepatitis B virus (HBV)-specific CD8^+^ T-cell response and the clinical outcome, in these patients. Although the majority of the participants undergoing Peg-IFN-α-2b therapy were non-responders, we observed a decrease in CD25 expression on their CD4^+^ T cells, suggesting that IFN-α therapy may provide a rationale for sequential IL-2 treatment without increasing regulatory T cells (Tregs). Following sequential therapy with IL-2, we demonstrated that the non-responders experienced a decrease in the numbers of Tregs and programmed cell death protein 1 (PD-1) expression. In addition, sequential IL-2 administration rescued effective immune function, involving signal transducer and activator of transcription 1 (STAT1) activation. Importantly, IL-2 therapy significantly increased the frequency and function of HBV-specific CD8^+^ T cells, which translated into improved clinical outcomes, including HBeAg seroconversion, among the non-responder CHB patients. Our findings suggest that sequential IL-2 therapy shows efficacy in rescuing immune function in non-responder patients with refractory CHB.

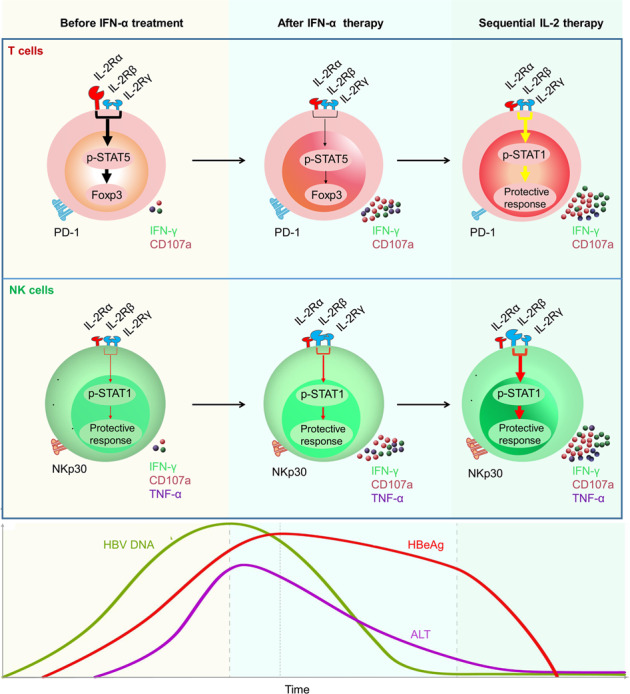

## Introduction

Although the Global Health Sector Strategy for the elimination of viral hepatitis by 2030 was approved at the 69th World Health Assembly, approximately 250 million patients, from 120 countries, are currently living with hepatitis B virus (HBV) infection.^1,2^ After the promising introduction of a highly effective treatment for hepatitis C virus (HCV) infections in 2013, hepatitis B was somewhat overshadowed by other public health priorities, and the possibility of a cure remained elusive.

To date, interferon alpha (IFN-α) represents the first-choice treatment for HBV infection.^3,4^ IFN-α is considered to exert antiviral effects through the activation of a series of interferon-stimulated genes (ISGs) and the degradation of nuclear viral DNA, likely via the exosomes or the apolipoprotein B editing complex 3 (APOBEC3) DNA-editing enzyme. In addition, IFN-α augments the immune function of cytotoxic CD8^+^ T cells, and rescues the cytotoxic activities of natural killer (NK) cells, which are important component of the innate antiviral immunity, and could respond rapidly to virus-infected cells and kill them.^5,6^ The quality and magnitude of cytotoxic CD8^+^ T-cell responses were important to overcome chronic virus infections;^7^ however, recent studies evaluating the efficacy of IFN-α therapy have reported a rate of hepatitis B e antigen (HBeAg) seroconversion of only 30%, and a rate of hepatitis B surface antigen (HBsAg) loss of ~5%.^8,9^ Therefore, CD8^+^ T cells alone may not be enough to clear the virus during persistent infection, and NK cells might be also worth paying attention to. Although IFN-α could activate T-cell responses during acute infection, chronic IFN-α exposure may drive immunosuppression and be detrimental to T cells that control pathogens.^10–12^ Wilson et al. reported that during persistent virus infection, chronic IFN-I signaling may drive the immunosuppressive program, and IFNR blockade dramatically improved T-cell immunity and control of LCMV infection.^13^ What’s more, we determined that IFN-α therapy for persistent HBV infection in humans may induce an immunomodulatory effect in chronic HBV patients by significantly upregulating levels of CD24^+^CD38^hi^ B cells, which could drive an immunosuppressive program and reduce antiviral effects.^14^ Suppressive mechanisms, such as the activity of regulatory T cells (Tregs) and programmed cell death protein 1 (PD-1) expression, have been demonstrated to occur during HBV infection.^15–18^ However, the modulation of these inhibitory pathways in vitro has led to effective functional recovery in only a minority of patients with chronic hepatitis B (CHB).^19^ Thus, the identification of new, well-tolerated therapies for the effective treatment of hepatitis B is critical.

Numerous recent studies have reviewed the multiple-functions of interleukin (IL)-2, such as its antiviral effects through the modulation of T cells and NK cells, as well as its role in the treatment of tumor.^20,21^ In addition, it has been shown in vitro that IFN-α improves IL-2 activity, thus exerting therapeutic effects in CHB.^22^ Combined IL-2 and IFN-α therapy has shown some promise in prolonging the survival of patients with RCC.^21^ However, IL-2 can have severe side effects when administered intravenously, and is known to drive Treg expansion.^23–26^ CD25 (IL-2Rα) is the “low-affinity” form of the IL-2 receptor (Kd~ 10^−8^ M). CD25-mediated downstream signaling induces forkhead box protein 3 (FOXP3) expression, which is indispensable for Treg production, through the phosphorylation of signal transducer and activator of transcription 5 (STAT5). The pairing of CD122 (IL-2Rβ) and CD132 (IL-2Rγ) subunits results in the formation of the intermediate-affinity IL-2 receptor (Kd = 10^−9^ M), which, when bound to IL-2, promotes the cytolytic activity and expansion of T cells. Furthermore, the binding of the CD25 to the intermediate-affinity receptor leads to the formation of the high-affinity receptor (Kd = 10^−11^ M),^27^ which is constitutively expressed at high levels on CD4^+^ Tregs, thus allowing them to compete with other types of cells for available IL-2.^20,28^ So far, few studies have demonstrated that IFN-α can inhibit the production of Tregs in the peripheral blood mononuclear cells (PBMC) of patients with CHB. In addition, it remains unclear whether sequential IL-2 therapy following IFN-α-2b therapy can improve the immune response in refractory CHB non-responder (NR) (α-2b) patients.

This study provides insight into the function of HBV-specific CD8^+^ T cells during sequential IL-2 therapy. Herein, we evaluated the expression of immunosuppressive molecules and the percentage of Treg cells in NR patients, and investigated HBV-specific CD8^+^ T-cell responses during sequential low-dose IL-2 therapy. Moreover, we aimed to ascertain whether this approach could improve the clinical outcome of NR patients who failed initial therapy with IFN-α.

## Results

### The proportion of CD25^+^CD4^+^ T cells is reduced following Peg-IFN-α-2b therapy

**Fig. 1:**
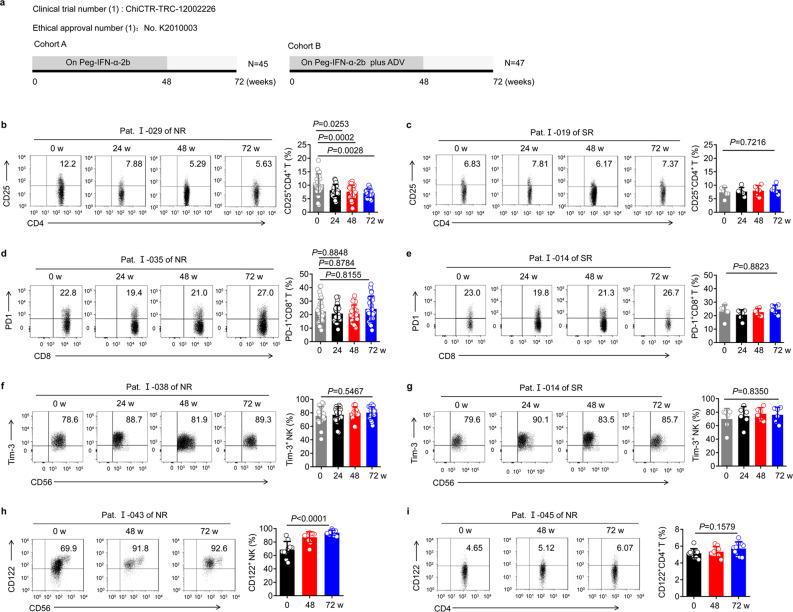
Reduced CD25 expression on CD4^+^ T cells after Peg-IFN-α-2b therapy. **a** The schematic representation of the study flow of the first clinical trial. **b**–**g** Data are analyzed and quantified for the peripheral blood mononuclear cells (PBMCs) of non-responder (NR) (α-2b) patients (*n* = 26) and seroconversion response (SR) patients (*n* = 6) at 72 weeks (48 weeks of Peg-IFN-α-2b + ADV therapy and 24 weeks of follow-up) from the start of therapy. Flow cytometry showing sequential proportion of CD25^+^CD4^+^ T cells from PBMCs of NR (α-2b) patients (**b**) and SR patients (**c**) over the course of the treatment; sequential PD-1 expression on CD8^+^ T cells from PBMCs of NR (α-2b) patients (**d**) and SR patients (**e**) during therapy; sequential Tim-3 expression on NK cells from PBMCs of NR (α-2b) patients (**f**) and SR patients (**g**) during therapy. For **b** to **g**, left: representative density plots of a representative sample. Right: statistical analysis of data from all samples. **h**, **i** Representative density plots (left) and percentage analysis (right) of CD122^+^ NK cells (**h**), and CD122^+^CD4^+^ T cells (**i**) from PBMCs of NR (α-2b) patients over the course of the treatment. *n* = 10. Data are analyzed by one-way ANOVA with Tukey’s multiple comparisons test. *P < 0.05; **P < 0.01; ***P < 0.001; ****P < 0.0001, and presented as mean ± SD

In the first clinical trial (Fig. 1a) we found that 30.4% of patients with CHB exhibited a serological response (SR) after therapy, with no significant difference (*P* = 0.5010) between group 1 (Peg-IFN-α-2b alone, 26.7%) and group 2 (Peg-IFN-α-2b + ADV, 34.0%) (Supplementary Table 1), which is consistent with previous reports.^29^ Thus, the majority of patients failed to achieve HBeAg loss. We subsequently chose to focus on these NR (α-2b) patients, and systematically analyzed the changes in their immune responses during treatment. We found that the proportion of CD25^+^CD4^+^ T cells (gated as shown in Supplementary Fig. 1a) was significantly reduced in NR (α-2b) patients, but not in SR patients, after the administration of Peg-IFN-α-2b at week 72 (72 w- 48 weeks of Peg-IFN-α-2b + ADV therapy and 24 weeks of follow-up) (Fig. 1b, c). Furthermore, CD25 expression on NK cells or CD8^+^ T cells was not significantly reduced in NR (α-2b) patients after Peg-IFN-α-2b therapy (Supplementary Fig. 2a, b). We then demonstrated that the expression of inhibitory molecules PD-1 on CD8^+^ T cells and T-cell immunoglobulin and mucin-domain containing-3 (Tim-3) on NK cells showed no significant changes at week 72 in both NR (α-2b) patients and SR patients (Fig. 1d–g).

Furthermore, the administration of Peg-IFN-α-2b markedly increased the proportion of CD122^+^ NK cells, but did not affect the proportion of CD122^+^CD4^+^ T cells or CD122^+^CD8^+^ T cells of NR (α-2b) patients (Fig. 1h, i; Supplementary Fig. 2c). However, little change in CD132 expression was detected during Peg-IFN-α-2b therapy (Supplementary Fig. 2d, e). Liao et al. and Siegel et al. reported that intermediate-affinity IL-2 receptor mediates positive immune signaling, and CD25 could convert intermediate-affinity receptor to high-affinity receptors, which are constitutively expressed on Treg cells, thereby inducing FOXP3 expression through STAT5 activation and finally mediating negative immune signaling.^30,31^ Collectively, these results suggested that after Peg-IFN-α-2b therapy, the proportion of CD25^+^CD4^+^ T cells decreased while the proportion of CD122^+^ NK cells increased; this resulted in the switching of IL-2R to mainly positive immune signaling, thus providing suitable conditions for sequential IL-2 therapy.

### IL-2 does not increase the expression of immunosuppressive molecules ex vivo

Based on our observations of a reduction in CD25 expression in NR (α-2b) patients after Peg-IFN-α-2b therapy, and the evidence from many studies indicating that antiviral immune responses are severely depressed in patients with CHB,^19^ we attempted to determine whether IL-2 plays a role in modulating NR (α-2b) patient immune cell effector function. IL-2 was initially recognized for its role in T-cell activation and proliferation, as well as in promoting the cytolytic activity of NK cells.^32^ Furthermore, IL-2 has been used to enhance antiviral T-cell responses in vivo^33,34^ and treat patients with RCC or melanoma.^27^ Twenty years ago, an attempt to use the combination of IFN-α-2b and IL-2 as a treatment approach for HBV was reported, with pronounced side effects, limited response rates, and an unsatisfactory decline in HBeAg levels.^33,35,36^ Given the safety risks and the limited benefits to patients reported in this study, we decided to first investigate the effectiveness of IL-2 ex vivo.

Considering the sustained effect of IFN-α therapy, after the administration of Peg-IFN-α-2b at treatment week 72, we stimulated PBMCs from NR (α-2b) patients with a very low dose of IFN-α,^37,38^ with or without IL-2. The findings showed that the expression of neither PD-1 nor Tim-3 was elevated in NR (α-2b) patients after IL-2 stimulation (Supplementary Fig. 3a, b, c). Interestingly, we found that the use of IL-2 only minimally affected the percentage of CD25^+^ CD4^+^ FOXP3^+^ Tregs ex vivo (gated as shown in Supplementary Fig. 1), regardless of the addition of transforming growth factor beta1 (TGF-β1, added at 3 ng/mL) to more closely mimic the in vivo environment in patients with CHB (Supplementary Fig. 3c, d). Furthermore, we showed that CD38 was significantly upregulated on both T cells and NK cells after IL-2 stimulation (Supplementary Fig. 3e-h). Thus, our findings indicate that IL-2 stimulation significantly promotes the activation of T cells and NK cells in NR (α-2b) patients ex vivo, without increasing the percentage of Tregs. These preliminary findings paved the way for the application of IL-2 in the in vivo aspect of our study.

### IL-2 promotes the secretion of IFN-γ from lymphocytes derived from liver biopsies of patients

**Fig. 2:**
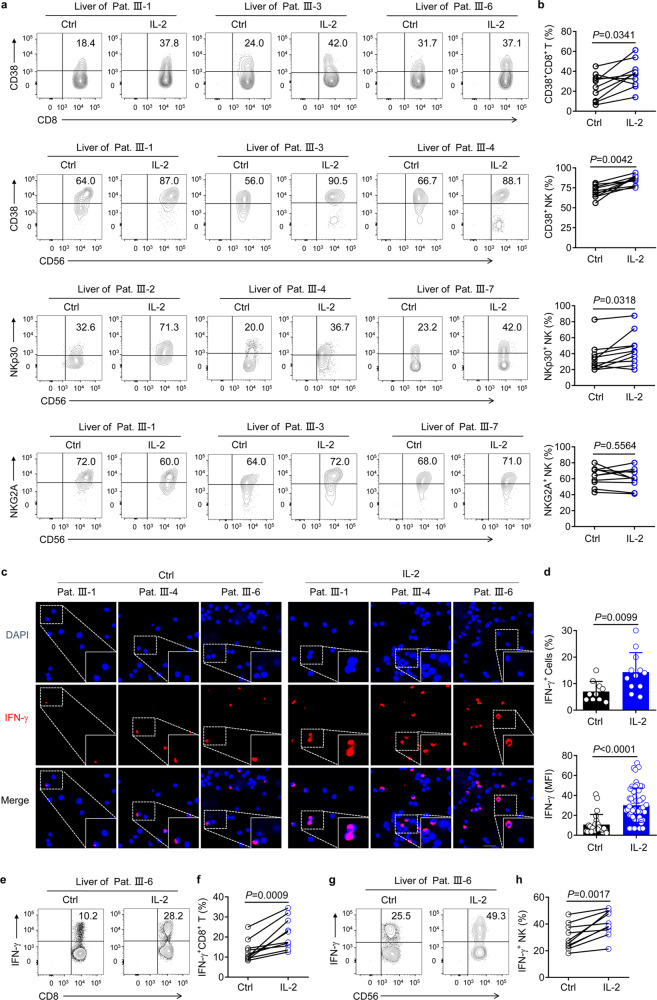
Improvement in the effector functions of lymphocytes derived from the liver biopsies of CHB patients, after IL-2 treatment ex vivo. Mononuclear cells were isolated from liver biopsies of patients with CHB, and stimulated with or without IL-2 (500 U/mL) overnight. **a**, **b** Representative density plots (**a**) and quantification (**b**) of CD38, NKp30, and NKG2A expression in gated CD8^+^ T cells and NK cells from liver biopsies, after IL-2 stimulation; *n* = 10. **c** Confocal microscopy analysis of IFN-γ expression in mononuclear cells from CHB patient liver biopsies, after IL-2 stimulation overnight, followed by treatment with monensin for 4 h. Scale bar = 20 µm. **d** (Upper) Statistics were based on the percentage of IFN-γ^+^ mononuclear cells isolated from liver biopsies per field of each group; each dot represents the percentage of IFN-γ^+^ mononuclear cells in one field. The total number of fields counted in each group without (Ctrl) and with IL-2 stimulation was 10 and 12, respectively. (Lower) Statistics were based on the MFI of IFN-γ from randomly selected single cell from each group; each dot represents the MFI of IFN-γ in one cell. The total number of IFN-γ^+^ cells in each group without and with IL-2 stimulation was 24 and 53, respectively. **e**–**h** Representative flow cytometry plots (**e**, **g**) and pooled data (**f**, **h**) were presented showing frequencies of IFN-γ^+^CD8^+^ T cells (**e**, **f**) and IFN-γ^+^ NK cells (**g**, **h**) in mononuclear cells isolated from liver biopsies, after being stimulated without or with IL-2 overnight, followed by being stimulated with PMA for 4 h. Data are analyzed by two-tailed paired Student’s *t* test (**b**, **f**, **h**), and two tailed unpaired Student’s *t* test (**d**); *P < 0.05; **P < 0.01; ***P < 0.001; ****P < 0.0001. Data are presented as mean ± SD

To further investigate whether IL-2 directly promotes the effector functions of lymphocytes in the liver, given the ethical considerations limiting such experimentation in patients, we performed liver biopsies before initiating IL-2 therapy. After stimulation with IL-2 ex vivo, the expression of CD38 and NKp30 on CD8^+^ T cells and NK cells, derived from liver biopsies, was significantly upregulated while NKG2A expression showed no significant change (Fig. 2a, b). In addition, immunofluorescence analyses demonstrated that IFN-γ expression was considerably increased in the lymphocytes obtained from liver biopsies of patients with CHB following IL-2 stimulation (Fig. 2c, d). Furthermore, we also demonstrated that the proportion of IFN-γ^+^CD8^+^ T cells as well as IFN-γ^+^ NK cells, derived from liver biopsies, increased significantly after IL-2 stimulation (Fig. 2e–h). Although we could not get intrahepatic T cells from non-HBV patients, these results suggested that IL-2 also improved the immune function of lymphocytes obtained from the liver biopsies of patients with CHB.

### IL-2 therapy does not promote Treg expansion or PD-1 expression in NR (α-2b) patients in vivo

**Fig. 3:**
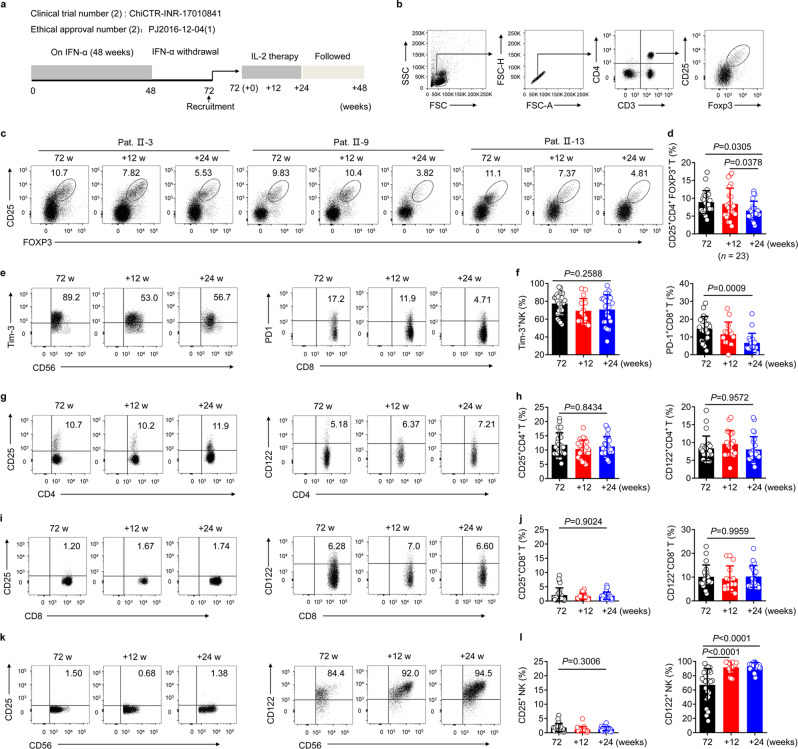
Reduction in the expression of immunosuppressive molecules after IL-2 therapy in vivo. **a** Schematic representation of the study flow second clinical trial. **b**–**l** Data obtained from the PBMCs of NR (α-2b) patients (*n* = 23) were quantified at week 72 (72 w; 48 weeks of IFN-α-2b therapy and 24 weeks of follow-up) from the start of therapy, and after 12 (+12 w) and 24 weeks (+24 w) of sequential IL-2 therapy. **b** Gating strategy for Tregs within PBMC samples. **c**, **d** Representative density plots (**c**) and pooled data (**d**) showing the percentage of Tregs within the PBMCs during IL-2 therapy. Representative density plots (**e**) and quantification (**f**) showing sequential Tim-3 expression on NK cells (**e**, **f**, left), and sequential PD-1 expression on CD8^+^ T cells (**e**, **f**, right), during IL-2 therapy. FACS analysis of the sequential proportion of CD25^+^CD4^+^ T cells (**g**, **h**, left) and the proportion of CD122^+^CD4^+^ T cells (**g**, **h**, right) during IL-2 therapy. FACS analysis of the sequential proportion of CD25^+^CD8^+^ T cells (**i**, **j**, left) and the proportion of CD122^+^CD8^+^ T cells (**i**, **j**, right) after IL-2 therapy. FACS analysis of the sequential proportion of CD25^+^NK cells (**k**, **l**, left) and the proportion of CD122^+^ NK cells (**k**, **l**, right) during IL-2 therapy. Data are analyzed by one-way ANOVA with Tukey’s multiple comparisons test (**d**, **f**, **h**, **j**, **l**); *P < 0.05; **P < 0.01; ***P < 0.001; ****P < 0.0001. Data are presented as mean ± SD

After we confirmed that IL-2 may improve the immune function of liver lymphocytes and not increase the percentage of CD25^+^ CD4^+^ FOXP3^+^ Tregs of PBMC ex vivo, we performed a second clinical trial with NR (α-2b) patients whose serum HBeAg showed no significant decline at week 72 (72 w; 48 weeks of IFN-α-2b therapy and 24 weeks of follow-up) after the start of IFN-α therapy to explore sequential IL-2 therapy effect. At 12 weeks after week 72, these patients were randomly allocated to treatment groups and were then treated with 1 × 10^6^ IU IL-2, twice weekly for 24 weeks (+24 weeks) (Fig. 3a and Supplementary Table 2). During the treatment period, patients were given ETV treatment, which has been shown to elicit a similar rate of HBeAg clearance or HBeAg seroconversion as treatment with either adefovir or lamivudine.^19^ In total, 23 patients completed the treatment. We then conducted a set of experiments to determine the safety and effectiveness of the therapy. Notably, no serious adverse events were observed (Supplementary Table 3). Furthermore, no bacterial infections were detected and none of the patients required antibiotic treatment.

Meng et al. showed that IL-2 therapy can downregulate PD-1 via the restoration of FBXO38 expression;^31^ similarly, we found that the frequency of Tregs and the expression of PD-1 decreased after sequential IL-2 therapy (+24 w) (Fig. 3b–f). This was also consistent with the findings by Wilson et al. in the context of LCMV infection.^9^ Analysis of IL-2R expression revealed an increase in the expression of CD122, but not in that of CD25 at 24 w post IL-2 therapy initiation (+24 w) in NK cells (Fig. 3g–l). Furthermore, we found that the percentage of Tregs and the expression of PD-1 on CD8^+^ T cells was not markedly altered in NR patients without sequential IL-2 therapy in the first clinical trial at 72 w and 96 w (+24 w) from the start of IFN-α therapy. Moreover, these patients showed no significant changes in IL-2R expression (Supplementary Fig. 4). Taken together, these findings indicate that IL-2 therapy does not rescue the expression of inhibitory molecules in vivo (in NR (α-2b) patients) and can be considered safe.

### IL-2 therapy promotes STAT1 activation in NR (α-2b) patients in vivo

**Fig. 4:**
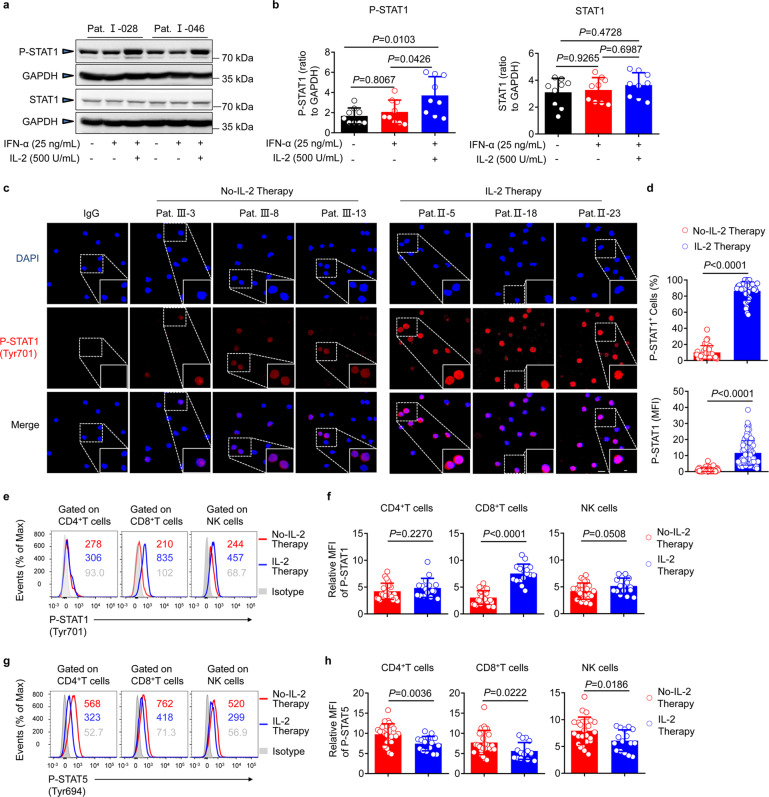
Increased P-STAT1 and decreased P-STAT5 expression after sequential IL-2 therapy in vivo. **a** Western blotting analysis of P-STAT1 and STAT1 levels in PBMCs of NR (α-2b) patient at week 72 (72 w; 48 weeks of Peg-IFN-α-2b therapy and 24 weeks of follow-up). PBMCs were stimulated with IFN-α (25 ng/mL) or co-stimulated with IL-2 (500 U/mL) for 30 min ex vivo. **b** Graphs on the right show the densitometric evaluation of **a**, confirming their significance; *n* = 9. **c** Confocal microscopy of P-STAT1 expression in the PBMCs of patients, with or without sequential IL-2 therapy for 24 weeks. **d** (Upper) Statistics were based on the percentage of P-STAT1^+^ PBMCs per field of each group; each dot represents the percentage of P-STAT1^+^ PBMCs in one field. The total number of fields counted in each group without (No-IL-2) and with sequential IL-2 therapy was 36 and 37, respectively. (Lower) Statistics were based on the MFI of P-STAT1 from randomly selected single cell from each group; the total number of P-STAT1^+^ PBMCs in each group without (No-IL-2) and with sequential IL-2 therapy was 64 and 113, respectively. Each dot represents the MFI of P-STAT1 in one cell. **e**, **f** Representative histograms (**e**) and relative MFI (**f**) of P-STAT1 expression in gated CD4^+^ T cells (left), CD8^+^ T cells (middle), and NK cells (right) within the PBMCs of NR (α-2b) patients, without (red, *n* = 26) or with (blue, *n* = 16) sequential IL-2 treatment for 24 weeks. **g**, **h** Representative histograms (**g**) and relative MFI (**h**) of P-STAT5 expression in gated CD4^+^ T cells (left), CD8^+^ T cells (middle), and NK cells (right) within the PBMCs of NR (α-2b) patients, without (red, *n* = 26) or with (blue, *n* = 16) sequential IL-2 treatment for 24 weeks. Data are analyzed by one-way ANOVA with Tukey’s multiple comparisons test (**b**), or two tailed unpaired Student’s *t* test (**d**, **f**, **h**); *P < 0.05; **P < 0.01; ***P < 0.001; ****P < 0.0001. Data are presented as mean ± SD

The STAT1 transcription factor is an important component of the classical JAK-STAT signaling pathway, and, consequently, IFN-based therapy, and is responsible for initiating the transcription of a large number of interferon-stimulated genes (ISGs).^39^ Typically, STAT1 can also be transferred by IL-2 signaling, and its phosphorylation (P-STAT1) is required for effector cell function. In the context of the antiviral response, STAT1 activation may promote the synergistic activity of IFN-α and IL-2. Herein, we found that the levels of active STAT1 (P-STAT1) were significantly increased after IL-2 stimulation ex vivo (Fig. 4a, b). Additionally, through immunofluorescence analyses and flow cytometry analysis, we demonstrated that P-STAT1 levels, particularly in CD8^+^ T cells, were significantly increased in vivo after IL-2 therapy at week 24 (Fig. 4c–f). Although IL-2/CD25 engagement could induce the phosphorylation of STAT5 (P-STAT5), which regulates FOXP3 expression,^40^ herein we found the amount of P-STAT5 decreased following exposure to IL-2, particularly in CD4^+^ T cells (Fig. 4g, h). These findings indicated that IL-2 plays a positive role in the activation of STAT1, which further highlights the potential opportunities for IL-2 therapy.

### IL-2 therapy increases the expansion and responses of HBV-specific CD8^+^ T cells in NR (α-2b) patients in vivo

**Fig. 5:**
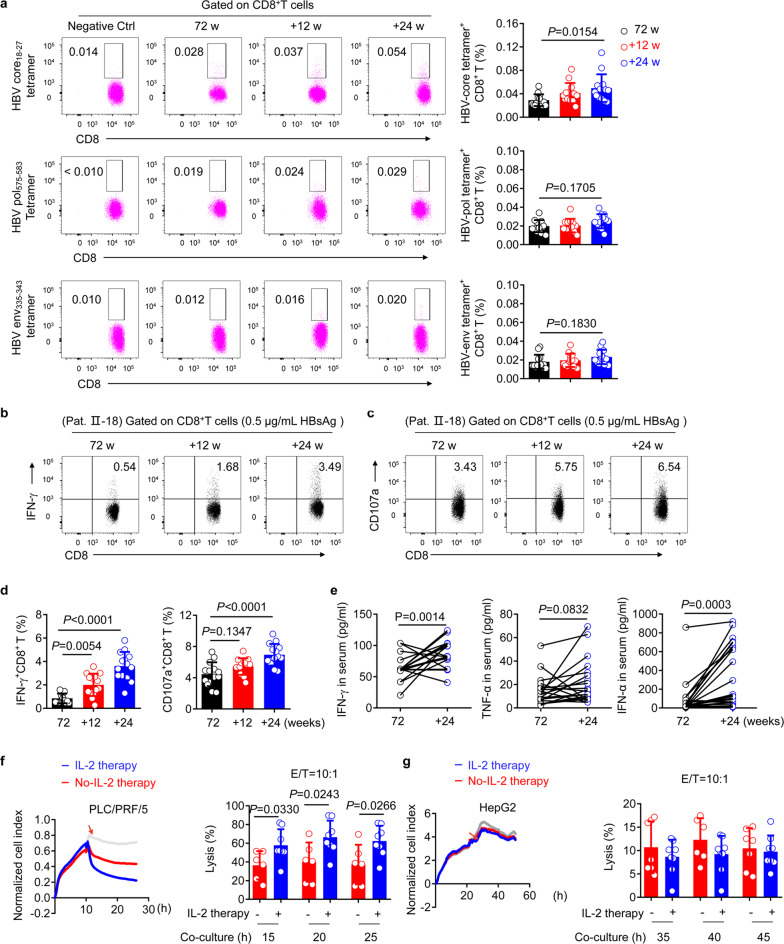
Augmentation of HBV-specific CD8^+^ T-cell immune responses and cytotoxicity after sequential IL-2 therapy in vivo. **a** Representative density plots (left panel) and pooled data (right panel) showing HBV core_18-27_-specific (upper), pol_575-583_-specific (middle), and env_335-343_-specific (lower) CD8^+^ T cells gated on live CD3^+^ T cells from PBMCs of NR patients at week 72 (72 w; 48 weeks of IFN-α-2b therapy and 24 weeks of follow-up), and after sequential IL-2 therapy for 12 (+12 w), and 24 (+24 w) weeks; *n* = 13. **b**, **c** PBMCs were treated with HBsAg (0.5 μg/mL) for 3 days. A representative image of intracellular cytokine staining is presented showing IFN-γ (**b**) and CD107a (**c**) expression on CD8^+^T cells. **d** Pooled data relating to the analyses of (**b**) and (**c**); *n* = 14. **e** IFN-γ (left), TNF-α (middle), and IFN-α (right) levels in serum were detected by ELISA at 72 w (black), and at the end of 24 weeks (+24 w) of sequential IL-2 therapy (blue); *n* = 23. **f** Time-dependent dynamic curve of the cell index (left) obtained by real-time cell analysis for PLC/PRF/5 cells after co-culture with purified CD8^+^ T cells from the PBMCs of patients (effector to target ratio (E/T) = 10:1), with (*n* = 8) or without (*n* = 6) IL-2 therapy for 24 weeks. Quantification (right) of CD8^+^ T-cell cytotoxicity measured with a real-time cell analyzer (RTCA). **g** Time dynamic curve of the cell index (left) obtained by RTCA for HepG2 cells under the same conditions as **f**, and quantification (right) of CD8^+^ T-cell cytotoxicity. Data are analyzed by one-way ANOVA with Tukey’s multiple comparisons test (**a**, **d**) or two-way ANOVA (**f**, **g**), or two-tailed paired Student’s *t* test (**e**); *P < 0.05; **P < 0.01; ***P < 0.001; ****P < 0.0001. Data were presented as mean ± SD

The number and function of HBV-specific CD8^+^ T cells are indispensable in HBV control,^41–45^ despite their low frequency in the majority of patients with CHB.^46,47^ To further investigate the effect of sequential IL-2 therapy in NR (α-2b) patients, the presence of circulating HBV-specific CD8^+^ T cells was detected by tetramer staining in HLA-A2^+^ patients. We found that the frequency of HBV core_18-27_ tetramers^+^ CD8^+^ T cells was significantly increased at the +24 w timepoint, following IL-2 therapy initiation. In addition, no change was observed in the frequency of HBV pol_575-583_ tetramers^+^ CD8^+^ T cells or HBV env_335-343_ tetramers^+^ CD8^+^ T cells during the treatment (Fig. 5a). However, in NR (α-2b) patients without sequential IL-2 therapy, no increase in the frequency of HBV-specific CD8^+^ T cells was observed (Supplementary Fig. 5a–c).

Next, to investigate the HBV-specific responses of CD8^+^ T cells, we examined the function of CD8^+^ T cells in the response to HBsAg in NR patients with or without sequential IL-2 therapy. PBMC of these patients were stimulated with HBsAg.^14,48^ We found that the proportions of IFN-γ^+^ CD8^+^ T cells and CD107a^+^ CD8^+^ T cells were significantly increased at the +24 w timepoint, following IL-2 therapy initiation (Fig. 5b–d). Furthermore, we found that serum levels of IFN-γ and IFN-α were markedly elevated, while serum levels of TNF-α rose only mildly at the end of IL-2 treatment (Fig. 5e).

To determine whether an increase in effector functions occurred without sequential IL-2 therapy, we then tested PBMCs from NR (α-2b) patients of the first clinical trial at week 96 (+24 w; 24 weeks of No-IL-2 therapy) from the start of IFN-α therapy. However, among these patients, who did not receive IL-2 therapy, we found that neither the proportion of IFN-γ^+^ CD8^+^ T cells nor that of CD107a^+^ CD8^+^ T cells had increased at the +24 w timepoint; this was accompanied by stable levels of serum IFN-γ, IFN-α, and TNF-α (Supplementary Fig. 5d–f). Moreover, after IL-2 therapy, we detected a significantly higher percentage of CD44^high^ CD62L^low^ effector memory CD8^+^ T (TEM) cells and increased expression of NKp30, which is reportedly associated with NK cell cytotoxicity.^49^ However, no significant changes in the percentage of TEM or NKp30 expression were observed in the IL-2-untreated group at the +24 w timepoint (Supplementary Fig. 5g, h; Supplementary Fig. 6a, b). To examine the dynamics of the functional modifications of NK cells after IL-2 treatment, we stimulated PBMC of NR (α-2b) patients with PMA, and demonstrated that the proportions of IFN-γ^+^ CD107a^+^ NK cells and IFN-γ^+^ TNF-α^+^ NK cells were all significantly elevated after 24 weeks of IL-2 therapy (+24 w), but were not markedly altered in the IL-2-untreated group (Supplementary Fig. 6c–f). Polyfunctional effector CD8^+^ T cells and NK cells are the main drivers of the antiviral response in patients with CHB;^50^ therefore, these findings showed that sequential IL-2 therapy could be beneficial to NR (α-2b) patients in whom IFN-α therapy has failed, by expanding these immune cell subsets.

Using a real-time cell analyzer (RTCA), we found that primary CD8^+^ T cells purified from the PBMCs of patients with CHB treated with IL-2 for 24 weeks caused cell death in the hepatocellular carcinoma (HCC) cell line PLC/PRF/5 more efficiently than the CD8^+^ T cells of patients who were not subjected to IL-2 therapy. Of note, the PLC/PRF/5 cell line shows persistent and stable HBsAg production, with physical integration of HBV DNA into its genome.^3^ However, the CD8^+^ T cells were not very efficient at killing HepG2 cells (Fig. 5f, g). Taken together, our findings demonstrate that sequential IL-2 therapy improves the expansion and responses of HBV-specific CD8^+^ T cells in NR (α-2b) patients with refractory CHB.

### IL-2 therapy improves the clinical outcome in refractory NR patients

**Fig. 6:**
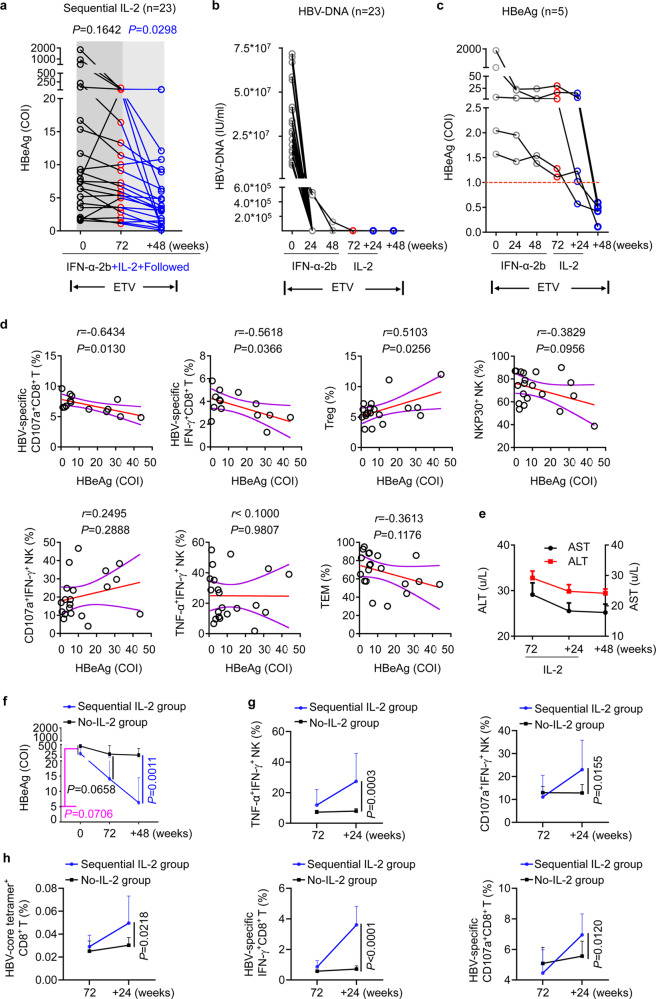
Improvement in clinical outcomes after sequential IL-2 therapy in vivo. **a**, **b** Cumulative data showing serum HBeAg levels (COI, cut-off index) (**a**) and HBV DNA levels (**b**) through IFN-α therapy (72 weeks; 48 weeks of IFN-α-2b + ETV therapy and 24 weeks of follow-up), sequential IL-2 therapy and follow-up (+48 weeks). *n* = 23. Data are analyzed by Wilcoxon matched-pairs signed rank test. **c** Dynamic curves indicating HBeAg levels for five patients who achieved HBeAg clearance through treatment. **d** Spearman’s rank correlation coefficient comparing HBeAg levels and the proportion of the indicated molecules at the end of the 24-week sequential IL-2 therapy. The Spearman correlation coefficient (*r*) and *P* value were shown. **e** The alanine aminotransferase (ALT; red) and aspartate aminotransferase (AST; black) levels of patients during and after IL-2 therapy. **f** The serum HBeAg levels of patients with (blue; *n* = 23) or without (black; *n* = 15) sequential IL-2 therapy were shown. **g**, **h** The proportions of TNF-α^+^IFN-γ^+^ NK cells, CD107a^+^IFN-γ^+^ NK cells (**g**), HBV core_18-27_ tetramers^+^ CD8^+^ T cells, HBV-specific IFN-γ^+^ CD8^+^ T cells and CD107a^+^ CD8^+^ T cells (**h**) in the sequential IL-2 group and the no-sequential IL-2 group at 72 weeks and +24 weeks (+24 w) timepoints. Data are analyzed by Mann–Whitney U test for **f**, and two tailed unpaired Student’s *t* test for **g** and **h**; *P < 0.05; **P < 0.01; ***P < 0.001; ****P < 0.0001. Data are presented as mean ± SD

To ascertain whether sequential IL-2 therapy improves clinical outcomes in refractory NR (α-2b) patients, we analyzed the clinical characteristics of 23 NR (α-2b) patients who completed courses of IFN-α and IL-2 treatment. To date, a durable HBsAg loss could be used to define ‘functional HBV cure’, and the HBeAg seroconversion response is still generally used to judge the treatment response.^4,51^ We found that the serum HBeAg levels, which showed little changes at week 72, were significantly reduced at week 120 (+48 weeks; 24 weeks of sequential IL-2 therapy and 24 weeks of follow-up) (Fig. 6a). Additionally, owing to the continuous treatment with ETV, HBV DNA was undetectable (<300 IU/mL) (Fig. 6b). Importantly, it is worth mentioning that five patients experienced HBeAg clearance (HBeAg seroconversion responders) after sequential IL-2 therapy (Fig. 6c); in addition, one patient exhibited HBsAg clearance with anti-HBs development (data not shown). Furthermore, the Spearman’s rank correlation coefficient, used to compare the relationships between various immune indicators and HBeAg levels, showed that after 24 weeks of IL-2 therapy, the proportion of HBV-specific CD107a^+^ CD8^+^ T cells and IFN-γ^+^ CD8^+^ T cells correlated negatively with HBeAg levels, while the frequency of Tregs correlated positively with HBeAg levels (Fig. 6d). However, we did not find significant changes in AST or ALT after IL-2 treatment (Fig. 6e).

We next attempted to determine whether the decrease in HBeAg levels was sustained after IFN-α therapy without sequential IL-2 treatment. We tested the serum of NR (α-2b) patients in the first clinical trial, and found that although serum HBeAg levels were notably reduced in NR patients participating in the first clinical trial at 72 weeks after the initiation of Peg-IFN-α-2b therapy, the decline in the 48-week follow-up after week 72 (+48 weeks; week 120) was not significant. The levels of HBeAb, AST, or ALT in these NR patients did not change significantly either, and HBV DNA was undetectable in the 48-week follow-up after week 72 (Supplementary Fig. 7a–d). Furthermore, we compared the HBeAg levels and immunological characteristics between the sequential IL-2 group and the no-sequential IL-2 group in the second trial. We found the serum HBeAg levels were much lower in the sequential IL-2 group than in the no-sequential IL-2 group at the +48 weeks timepoint (Fig. 6f). In addition, the proportions of TNF-α^+^IFN-γ^+^ NK cells and CD107a^+^IFN-γ^+^ NK cells were higher in the sequential IL-2 group than in the no-sequential IL-2 group at the +24 weeks timepoint (+ 24 w; the peak of the immune response after IL-2 therapy) (Fig. 6g). Importantly, the patients in the sequential IL-2 group had higher frequency of HBV core_18-27_ tetramers^+^ CD8^+^ T cells, the proportions of HBV-specific IFN-γ^+^ CD8^+^ T cells and CD107a^+^ CD8^+^ T cells than the patients in the no-sequential IL-2 group (Fig. 6h). Collectively, the findings demonstrated that the administration of IL-2 to NR (α-2b) patients increased the rate of HBeAg seroconversion from 20–30% (after IFN-α therapy alone), to >50%. In addition, IL-2 therapy improved the effector functions of CD8^+^ T cells and NK cells without mediating the upregulation of immunosuppressive molecules, thereby eliciting a potent antiviral immune response.

## Discussion

In this study, we demonstrated that sequential IL-2 therapy rescues immune function in patients with refractory CHB. We show that, in most patients in whom IFN-α therapy failed, sequential IL-2 treatment achieves promising therapeutic effects in restoring the immune response and promoting the loss of HBeAg. Collectively, our findings make three important contributions to elucidating the underlying mechanisms and explaining the observed clinical outcomes following sequential IL-2 treatment.

First, we demonstrated the limited efficacy of IFN-α therapy and describe the rationale for the use of IL-2. Evaluation of 92 patients with CHB who had been treated with Peg-IFN-α-2b for 48 weeks revealed that approximately 70% were non-responders, i.e., patients who failed to achieve HBeAg loss. CD25 mediates the activation of STAT5 signaling, which is necessary for FOXP3 induction, particularly in CD4^+^ T cells. Furthermore, the binding of intermediate-affinity IL-2 receptor to IL-2 on CD8^+^ T cells and NK cells promoted their cytolytic activity and expansion via the activation of STAT1 signaling. However, intermediate-affinity IL-2 receptor can be converted into a high-affinity receptor via its association with CD25; this receptor is constitutively expressed in FOXP3^+^ Tregs, which suppress effector functions of a wide range of immune cells.^52^ IL-2/CD25 signaling is required for expanding Treg cells, which is a major concern for the therapeutic application of IL-2. Interestingly, we observed that the proportion of CD25^+^CD4^+^ T cells significantly decreased after Peg-IFN-α-2b therapy, suggesting that IFN-α therapy may provide a rationale for sequential IL-2 treatment without increasing Treg cells.

Second, we showed that IL-2 restored the immune response of NR patients both ex vivo and in vivo. We initially found that stimulation with IL-2 increased the expression of CD38 by both T cells and NK cells among the PBMCs of NR (α-2b) patients. Moreover, the proportion of Tregs within the PBMCs showed no increase, despite co-stimulation with TGF-β. Of note, IL-2 also promotes CD38, NKp30, and IFN-γ expression by CD8^+^ T cells and NK cells derived from liver biopsies of patients with CHB. Therefore, our data indicate that IL-2 represents a safe and effective candidate for the augmentation of immune effector functions in NR (α-2b) patients. However, there are some limitations to this study. For example, detection of P-STAT1/P-STAT5 or IL-2R expression in liver biopsies was not performed in a sufficiently large number of cells; furthermore, liver biopsies from patients after IL-2 therapy or patients without hepatitis B, or IL-2 treated patients with nonalcoholic fatty liver disease, were not used as controls.

To further verify the effectiveness of IL-2 in vivo, we conducted a second clinical trial in patients for whom IFN-α therapy had failed. The multiple immunosuppressive pathways that exist in the CHB environment were examined during IL-2 therapy. We demonstrated that after IL-2 therapy, the proportion of PD-1^+^ CD8^+^ T cells and Tregs was reduced. In addition, the expression of P-STAT5 in CD4^+^ T cells was significantly downregulated, whereas the expression of P-STAT1 was significantly upregulated. Importantly, sequential IL-2 therapy increased the frequency of HBV-specific CD8^+^ T cells. Further, the HBsAg-specific immune response of CD8^+^ T was also prominently restored after IL-2 therapy. Using RTCA, we showed that CD8^+^ T-cell cytotoxicity was also rescued. Thus, these findings indicate the potential therapeutic utility of IL-2 in the restoration of the antiviral immune response in NR (α-2b) patients.

Third, we demonstrated that sequential IL-2 therapy contributed to improved clinical outcomes. We investigated the levels of serological markers in 23 patients, and found that HBeAg levels in most patients were significantly reduced. Five patients showed HBeAg loss, and one patient exhibited HBsAg loss after IL-2 therapy. In addition, we demonstrated a significant correlation between HBeAg and the frequency of HBV-specific CD107a^+^ CD8^+^ T cells, as well as IFN-γ^+^ CD8^+^ T cells.

Despite the reported efficacy of combined IFN-α and IL-2 therapy, several important questions remain to be explored, predominantly those relative to the continued unsatisfactory clinical outcomes evident following IL-2 therapy. We speculated that the dose of IL-2 or duration of sequential IL-2 therapy may require further optimization. Moreover, although many patients did not show HBeAg clearance, a rescued immune response and effector functions were noted to some degree following sequential IL-2 therapy. Thus, we considered these patients may have the potential for HBeAg seroconversion. For these refractory NR(α-2b) patients, the identification of alternative novel treatment strategies is necessary. Furthermore, though it would be important and useful to comprehensively analyze the immune function of lymphocytes, and the tissue resident populations along with the infiltrating immune cells populations obtained from the liver biopsies of patients pre- and post-IL-2 therapy, it is difficult and is not feasible in the clinical setting. In addition, the development of appropriate mouse model systems that simulate the progression of CHB in humans remains a challenge. By extension, if FOXP3^+^ Tregs showed no increase in patients with CHB and in the tumor environment after pre-treatment, sequential IL-2 therapy (combined with immune checkpoint blockade) may have an application in the eradication of tumors such as hepatocellular carcinoma. Collectively, our results highlight a novel antiviral strategy, and demonstrate that sequential IL-2 therapy enhances the immune response of patients with refractory CHB and contributes to improved clinical outcomes.

## Materials and methods

### Human subjects and study design

Patients with CHB were selected from among participants in two studies. The trial registration numbers were ChiCTR-TRC-12002226 and ChiCTR-INR-17010841 (http://www.chictr.org.cn/), and the studies were approved by the local Ethics Board of the First Affiliated Hospital of Anhui Medical University (approval numbers: No. K2010003 and PJ2016-12-04(1)). Experiments were conducted in accordance with the ethical guidelines of the 1975 Declaration of Helsinki, the Principles of Good Clinical Practice, and the guidelines of China’s regulatory requirements.

Each participant provided written informed consent. Inclusion criteria were as follows: patients with CHB ranged in age from 18 to 65 years; prior to receiving IFN-α therapy, HBsAg- and HBeAg- positive for longer than 24 weeks, with detectable baseline serum HBV DNA (>2 × 10^4^ IU/mL). Furthermore, they had elevated serum alanine aminotransferase levels (ALT, >2 × upper limit of normal, ULN, and <10 × ULN) on at least two occasions. Exclusion criteria were as follows: prior antiviral or immunomodulatory therapy for CHB; hepatitis D viral infection, human immunodeficiency viral infection, or the presence of serum antibodies against HCV; liver cirrhosis; or other acquired or inherited causes of liver disease. Patients of this open-label study were randomly assigned to the groups by simple randomization with no stratification, and were assigned to a serial number. The number was linked to a computer-generated randomization list.

For clinical trial 1 (patients were represented as I-01, etc.), equivalent numbers of eligible patients were randomly divided into two groups. Group 1: patients were treated with 1.5 µg/kg/week Peg-IFN-α-2b (PegIntron, Schering-Plough, Kenilworth, NJ, USA) monotherapy, Group 2: patients were treated with Peg- IFN-α-2b in combination with adefovir (ADV, 10 mg/day, Hepsera, Gilead Sciences, Foster City, CA, USA), for 48 weeks, with 24 weeks of follow-up. All patients had received ADV during the 24 weeks of follow-up. The outcome observed was the rate of HBeAg seroconversion. We used the non-inferiority test to estimate the required sample size. According to the existing reports,^53,54^ we estimated the rate of HBeAg seroconversion was 0.24 in Group 1; and 0.40 in Group 2. *α* = 0.025, *β* = 0.2, and the power of the test was 0.8. The allocation ratio of the two groups was 1. *NIM* (Non-Inferiority Margin) was -0.1. The Power Analysis and Sample Size (PASS) software was used to estimate the sample size. Furthermore, to ensure that there was a sufficient number of patients to observe the effects after withdrawal from the study, the loss to follow-up rate was estimated to be approximately 10%, which indicated that 46 subjects were required for each group.

At last, a total of 92 patients were randomly allocated to the treatment groups and completed the treatment (Supplementary Table 1); among them, 28 were classified as seroconversion response (SR) patients owing to HBeAg loss (cut off index, i.e., COI < 1) and seroconversion to anti-HBeAg (HBV DNA < 2000 IU/mL) at the end of 72 weeks (72w, comprising 48 weeks of Peg-IFN-α-2b therapy and 24 weeks of follow-up). The remaining 64 patients that did not exhibit HBeAg clearance at the end of this 72-week-period were classified as non-responders (NR) (α-2b) patients.

For clinical trial 2 (patients were represented as II-01, etc.), all the patients received a dose of 5 × 10^6^ IU IFN-α-2b (AnkeBio Co. Ltd., China) every other day for 48 weeks, with 0.5 mg/day entecavir (ETV, Anhui Biochem United Pharmaceutical Co., Ltd), followed by 24 weeks of follow-up (72 weeks in total), and failed to achieve HBeAg loss (i.e., NR (α-2b) patients). Next, NR (α-2b) patients were randomly assigned to two groups given oral ETV daily alone (Group 1) or in combination with 1 × 10^6^ IU recombinant human IL-2 (Beijing Four Rings Biopharmaceutical. Co. Ltd., China) by intramuscular injection twice a week (Group 2), for 24 weeks, followed by a 24-week follow-up. The outcome observed was the rate of HBeAg seroconversion. We used superiority test to estimate the required sample size. According to the existing reports,^36,55,56^ we estimate the rate of HBeAg seroconversion was 0.04 in Group 1; and 0.50 in Group 2. *α* was 0.05, *β* was 0.2, and power of test was 0.8. The allocation ratio of the two groups (*N1*: *N2*) was 1:2. *NIM* was 0.1. The PASS software was used to estimate the sample size. Furthermore, the loss to follow-up rate was estimated to be approximately 10%, which indicated that 16 subjects were required for group 1, and 32 subjects were required for group 2.

At last, a total of 38 NR (α-2b) patients were randomly allocated to the groups and completed the treatment (Supplementary Table 2); among them, 23 NR (α-2b) patients were administered IL-2. All the patients received ETV throughout the study.

Patients with CHB who had not received IFN-α or IL-2 therapy were represented as III-1, etc. Liver biopsies were obtained from 10 patients with CHB who had not been subjected to IL-2 therapy, but had received ETV for 48 weeks.

### Analysis of HBV-specific CD8^+^ T cells

For clinical trial 2, thirteen patients of sequential IL-2 therapy group and nine patients of non-IL-2 therapy group were HLA-A2 positive (Supplementary Table 2). Synthetic peptides and the corresponding phycoerythrin (PE)-conjugated tetrameric peptide-HLA Class I complexes representing the HLA-A2 restricted HBV-derived epitopes (core_18-27_: FLPSDFFPSV; env_335-343_: WLSLLVPFV; pol_575-583_: FLLSLGIHL) were purchased from MBL Beijing Biotech Co., Ltd. The PBMC of these HLA-A2^+^ patients were incubated with tetramers at 25 °C for 30 min. The cells were then washed and stained with antibodies for surface markers in accordance with the manufacturer’s instructions. Data were collected using a BD LSRII (Becton Dickinson), and then analyzed with FlowJo software.

### Analysis of immune cell-mediated killing

A real-time cell analyzer (RTCA, XceLLigence, Roche) was used to perform real-time monitoring of CD8^+^ T-cell cytotoxicity. The CD8^+^ T cells were purified through magnetic-activated cell sorting (Miltenyi Biotec) from the PBMC of patients with CHB. The target cells, PLC/PRF/5 cells and HepG2 cells, were purchased from the Institute of Cell Research in Shanghai, China, and were tested negative for mycoplasma contamination. PLC/PRF/5 cells or HepG2 cells were plated in 16-well E plates (ACEA Biosciences) at a density of 1 × 10^4^ cells per well; CD8^+^ T cells were then added to the culture wells at a density of 1 × 10^5^ cells per well after stable adherent growth. Impedance data were collected over 25 h on an RTCA multi plate system.

### Isolation of cells from liver biopsy material and flow cytometry

For the isolation of liver infiltrating mononuclear cells from liver biopsies of patients with CHB, liver tissues were firstly dissected into small pieces, followed by mechanical disruption using cell scrapers (BD) in RPMI 1640 complete medium (10% fetal calf serum, and 1% streptomycin/penicillin). Next, debris were carefully removed by filtering through stainless steel meshes, followed by passing single-cell suspension through 70 µm cell strainers.^57^ After lysis of the red blood cells (RBC) using RBC lysis buffer (biolegend, # 420301), the mononuclear cells were obtained and stimulated with or without IL-2 (500 U/mL) overnight. Next, cell suspensions were surface-labeled for human antibodies (as described in Supplementary Table 4) for 30 min at 4 °C. Prior to staining with antibodies, mouse serum was used to block the binding of non-specific Fc-receptors. Homologous immunoglobulin G molecules were used as isotype control antibodies. For intracellular staining of IFN-γ, cells were stimulated with phorbol 12-myristate 13-acetate (PMA) (50 ng/mL; Sigma) and ionomycin (1 μg/mL; Calbiochem) in the presence of monensin (10 μg/mL; Sigma) for additional 4 h. Cells were fixed and permeabilized using a Foxp3/Transcription Factor Staining Buffer Set in accordance with the manufacturer’s instructions (eBioscience). Data were collected using the FCM LSR II flow cytometer (BD Biosciences, USA) and analyzed with FlowJo software (Tree Star, USA).

### Statistical analysis

We used two-tailed unpaired or paired Student’s *t* tests between two groups, one-way analysis of variance (ANOVA) across multiple groups, the Mann–Whitney U test for continuous variables, and Chi-Squared and Fisher exact tests for categorical variables, and the Wilcoxon signed rank test. We employed Prism 8 (GraphPad) software to determine statistical significance. Statistical parameters were indicated in the figure legends of each figure (*P < 0.05; **P < 0.01; ***P < 0.001; ****P < 0.0001). Data are presented as mean ± SD.

Other detailed methods are available in the Supplementary material.

## Supplementary information

Supplementary material

Supplementary Table 1

## Data Availability

The data used and/or analyzed to support the findings of this study are available in this paper or the Supplementary Information. Any other data that support the findings of this study are available from the corresponding author upon reasonable request.
