# Supplementary material for: Restoration of HBV-specific CD8+ T-cell responses by sequential low-dose IL-2 treatment in non-responder patients after IFN-α therapy
Source: Signal Transduct Target Ther. 2021 Nov 5;6:376. doi: 10.1038/s41392-021-00776-0 (PMC8569154; doi:10.1038/s41392-021-00776-0)
Supplement: Supplementary file 1 — Supplementary material [file 41392_2021_776_MOESM1_ESM.docx]

**Restoration of HBV-specific CD8^+^ T Cell Responses by Sequential Low-dose IL-2 Treatment in Non-responder Patients after IFN-α Therapy**

Dongyao Wang ^1,2,4^, Binqing Fu ^1,2,4^, Xiaokun Shen ^1,2^, Chuang Guo ^1,2^, Yanyan Liu ^3^, Junfei Zhang ^3^, Rui Sun ^1,2^, Ying Ye ^3^, Jiabin Li ^3^*, Zhigang Tian ^1,2^*, Haiming Wei ^1,2^*

1. Institute of Immunology and the CAS Key Laboratory of Innate Immunity and Chronic Disease, School of Basic Medicine and Medical Center, University of Science and Technology of China, Hefei, Anhui 230001, China.

2. Hefei National Laboratory for Physical Sciences at Microscale, University of Science and Technology of China, Hefei, Anhui 230001, China.

3. Department of Infectious Diseases, the First Affiliated Hospital of Anhui Medical University, Hefei, Anhui, 230027, China.

4. These authors contributed equally

**Supplementary Materials and Methods**

**Cell preparation and culture**

PBMCs were isolated from peripheral blood by Ficoll density gradient centrifugation and resuspended in RPMI 1640 complete medium (10% fetal calf serum, and 1% streptomycin/penicillin). The cell line, HepG2, was maintained in our laboratory. The cell line PLC/PRF/5 was a generous gift from Mian Wu (University of Science and Technology of China). Both cell lines were negative for mycoplasma contamination.

***Ex vivo* flow cytometry experiments**

Lymphocyte suspensions were stained with human monoclonal antibodies (as described in **Table S4**). Prior to staining with antibodies, mouse serum was used to block the binding of non-specific Fc-receptors. Intracellular staining of cytokines was performed on PBMC with 4 hours of stimulation with phorbol 12-myristate 13-acetate (PMA) (50 ng/mL; Sigma) and ionomycin (1 μg/mL; Calbiochem) in the presence of monensin (10 μg/mL; Sigma). Intracellular staining of HBsAg-specific cytokines was performed on PBMC with 3 days of stimulation with HBsAg (0.5 μg/mL; HyTest). Monensin and anti-CD107a was added at the same time for the final 4 h. Cells were then collected and stained for surface or intracellular markers in accordance with the manufacturer's instructions, using antibodies were purchased from BD Biosciences. Data were collected using the FCM LSR II flow cytometer (BD Biosciences, USA) and analyzed with FlowJo software (Tree Star, USA).

**Immunoﬂuorescence analysis**

PBMCs were ﬁxed in 4% paraformaldehyde and incubated with PBS supplemented with 5% goat serum and 0.5% Triton-X, at room temperature, for 1 h. Cells were then stained with Phospho-STAT1 (Tyr701) antibody (CST) overnight at 4 °C, followed by staining with goat anti-rabbit IgG (Invitrogen). For the detection of IFN-γ (BD), lymphocytes obtained by liver biopsy were first stimulated with IL-2 (500 U/mL) for 12 h, and then treated with monensin for 4 h. Cells were then stained with 4′,6-diamidino-2-phenylindole (DAPI). Images were visualized on a Zeiss 880 Meta multi-photon confocal microscope (Zeiss, Oberkochen, Germany).

**Western blotting**

After stimulation with IFN-α or IL-2, PBMCs were collected and lysed in a radioimmunoprecipitation assay (RIPA) buffer (Beyotime, China). After centrifugation at 14,000 *g* for 15 minutes to remove cell debris, the pellet was discarded. The protein concentration in the supernatant was then determined via a bicinchoninic acid assay (BCA, Pierce, USA). Western blots were performed according to the standard protocol. Protein samples (50 µg) were then loaded and separated using sodium dodecyl sulfate polyacrylamide gel electrophoresis (SDS-PAGE), before being transferred onto a 0.45 µm polyvinylidene fluoride membrane, blocked in 5% (w/v) bovine serum albumin (BSA), and incubated with primary antibodies. Following incubation, the samples were washed five times with wash buffer and horseradish peroxidase (HRP)-conjugated goat anti-rabbit IgG (Sangon Biotech, #D110058) or HRP-conjugated goat anti-rabbit IgG (Sangon Biotech, #D110087). Protein bands were then visualized and analyzed by chemiluminescence (Millipore). The primary antibodies (1:1000 dilution for STAT1 and pSTAT1 [Tyr701]) were obtained from Cell Signaling Technology, with the exception of anti-glyceraldehyde 3-phosphate dehydrogenase (GAPDH, used at 1:5000 dilution), which was purchased from Santa Cruz (USA).

**Enzyme-linked immunosorbent assay (ELISA)**

The IFN-γ, tumor necrosis factor (TNF)-α, and IFN-α levels in the sera of patients, before and after IL-2 treatment, were measured using ELISA kits (Dakewe Biotech Co., Ltd. Cat# 1110002, 1117202 and 1110012, respectively), in accordance with the manufacturer’s instructions.

**Supplementary Figures and Figure Legends**


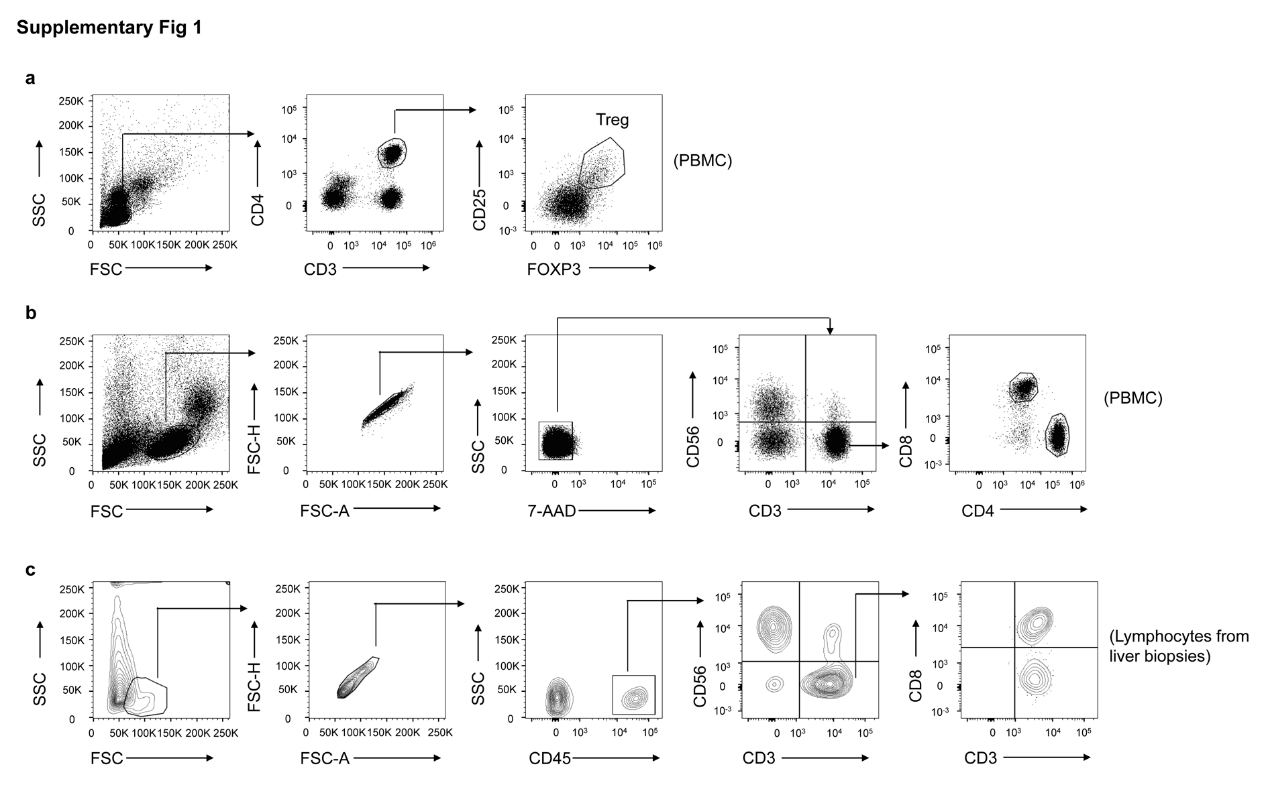


**Supplementary Fig. 1.** **Flow cytometry gating strategy.** **a.** Gating strategy for Tregs within peripheral blood mononuclear cell (PBMC) samples. **b.** Gating strategy for NK cells, CD8^+^ T cells, and CD4^+^ T cells, derived from PBMC samples. **c.** Gating strategy for NK cells and CD8^+^ T cells derived from patient liver biopsies.


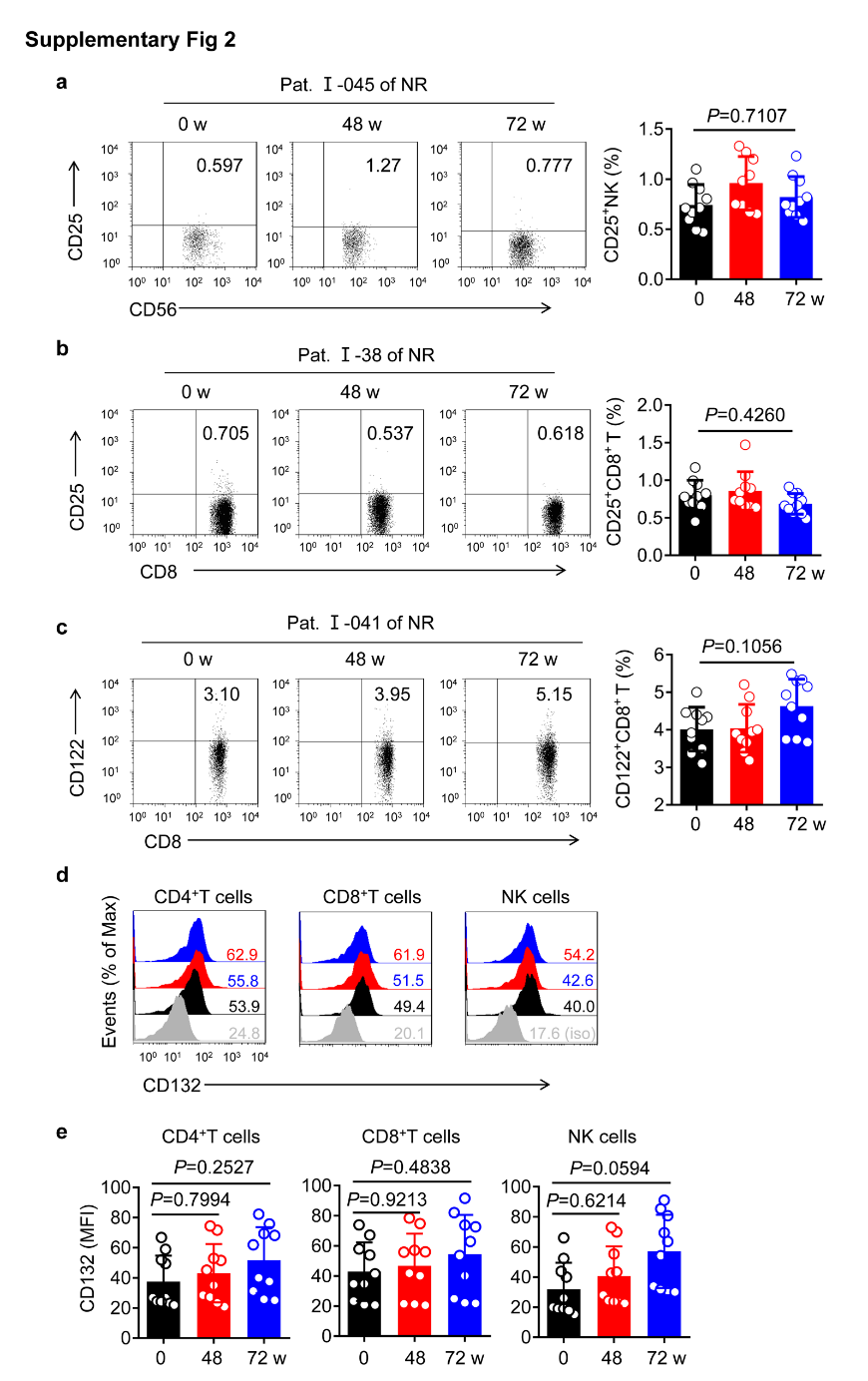


**Supplementary Fig. 2. CD25 and CD132 expression on CD8^+^T cells and NK cells were not increased after Peg-IFN-α-2b therapy.** Data are analyzed and quantified from the peripheral blood mononuclear cells (PBMCs) of non-responder (NR) (α-2b) patients (*n* = 10) at 72 weeks (48 weeks of Peg-IFN-α-2b + ADV therapy and 24 weeks of follow-up) from the start of therapy. **a, b.** Representative density plots (left) and percentage analysis (right) of sequential CD25 expression in NK cells **(a)** and CD8^+^ T cells **(b)** through the therapy. **c**. Representative density plots (left) and percentage analysis (right) of sequential CD122 expression in CD8^+^ T cells through the therapy. **d, e.** Representative histograms **(d)** and summarized quantification **(e)** of mean ﬂuorescence intensity (MFI) relating to the expression of CD132 on gated CD4^+^T cells (left), CD8^+^T cells (middle), and NK cells (right) through the therapy. All Data are analyzed by two-way ANOVA; **P* < 0.05; ***P* < 0.01; ****P* < 0.001; *****P* < 0.0001. Data are presented as mean ± SD.


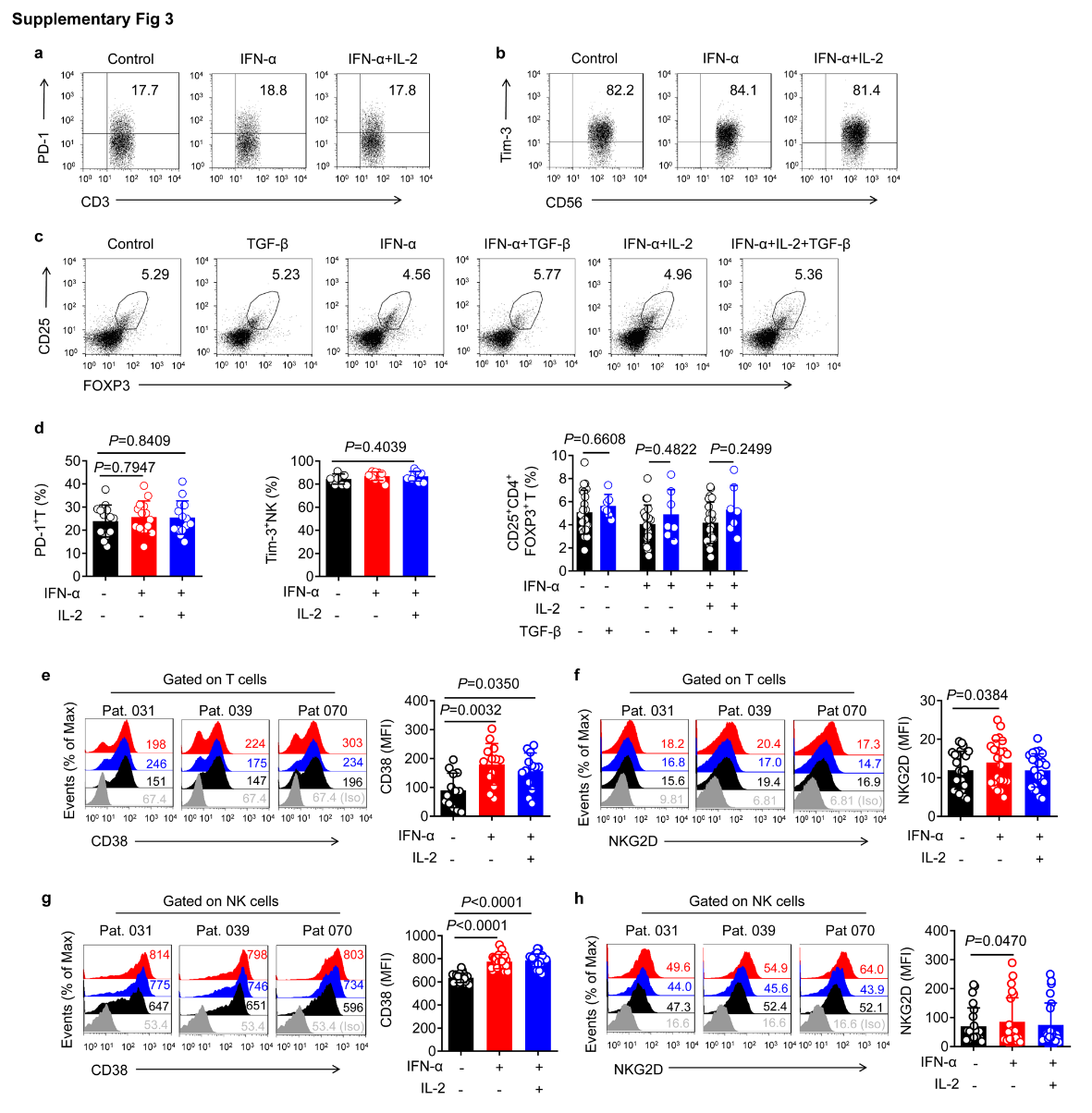


**Supplementary Fig. 3.** **IL-2 treatment *in vitro* did not increase the expression of immunosuppressive molecules, but promoted the activation of T cells and NK cells.** The PBMCs of NR (α-2b) patients (clinical trial 1) at week 72 (72 w; 48 weeks of Peg-IFN-α-2b + ADV therapy and 24 weeks of follow-up) were stimulated with IFN-α (25 ng/mL) or co-stimulated with IL-2 (500 U/mL) for 32 h. **a, b.** Representative density plots showing the analysis of PD-1 expression on CD8^+^ T cells **(a)**, *n* = 14; and Tim-3 expression on NK cells **(b)**, *n* = 10. **c.** Representative density plots showing the analysis of Tregs within NR (α-2b) patient PBMCs that were stimulated with IFN-α (25 ng/mL), and/or IL-2 (500 U/mL), and/or TGF-β1 (3 ng/mL). *n* = 7 for the additional TGF-β1 group, and *n* = 22 for the non-TGF-β1 group. **d.** Pooled data of each panel relating to the analyses of **(a), (b),** and **(c)**. **e, f.** Representative histograms (left) and summarized quantification (right) of MFI relating to the expression of CD38 **(e)** and NKG2D **(f)** on gated T cells. **g, h.** Representative histograms (left) and summarized quantification (right) of MFI relating to the expression of CD38 **(g)** and NKG2D **(h)** on gated NK cells. For **(e)** to **(h)**, *n* = 19. All Data are analyzed by one-way ANOVA with Tukey’s multiple comparisons test; **P* < 0.05; ***P* < 0.01; ****P* < 0.001; *****P* < 0.0001. Data are presented as mean ± SD.


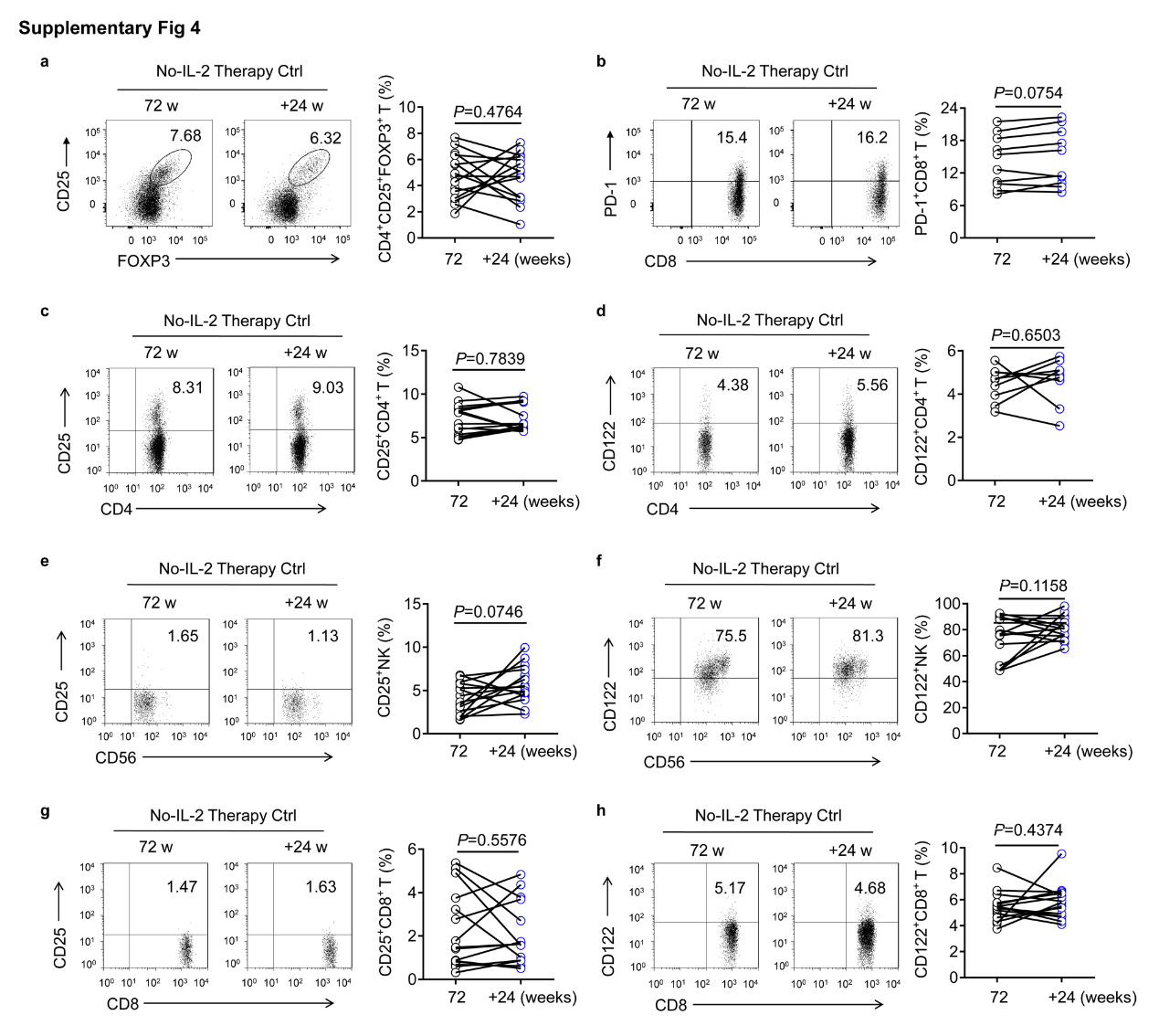


**Supplementary Fig. 4.** **The expression of immunosuppressive molecules did not change significantly in patients without sequential IL-2 therapy.** Data were derived for the PBMCs of NR (α-2b) patients at week 72 (72 w; 48 weeks of Peg-IFN-α-2b therapy and 24 weeks of follow-up) and week 96 (+24 w; a further 24 weeks without sequential IL-2 therapy). **a, b.** Representative density plots (left) and pooled data (right) showing the percentage of Tregs **(a)**, *n* = 18; and the expression of PD-1 on CD8^+^ T cells **(b)**, *n* = 10, within the PBMC samples. **c-h.** Representative density plots (left) and quantiﬁcation (right) of CD25 **(c)** and CD122 expression **(d)** in CD4^+^ T cells; CD25 **(e)** and CD122 expression **(f)** in NK cells; CD25 **(g)** and CD122 expression **(h)** in CD8^+^ T cells. For **(c)** to **(h)**, *n* = 15. All Data are analyzed by two-tailed paired Student’s t-test; **P* < 0.05; ***P* < 0.01; ****P* < 0.001; *****P* < 0.0001. Data are presented as mean ± SD.


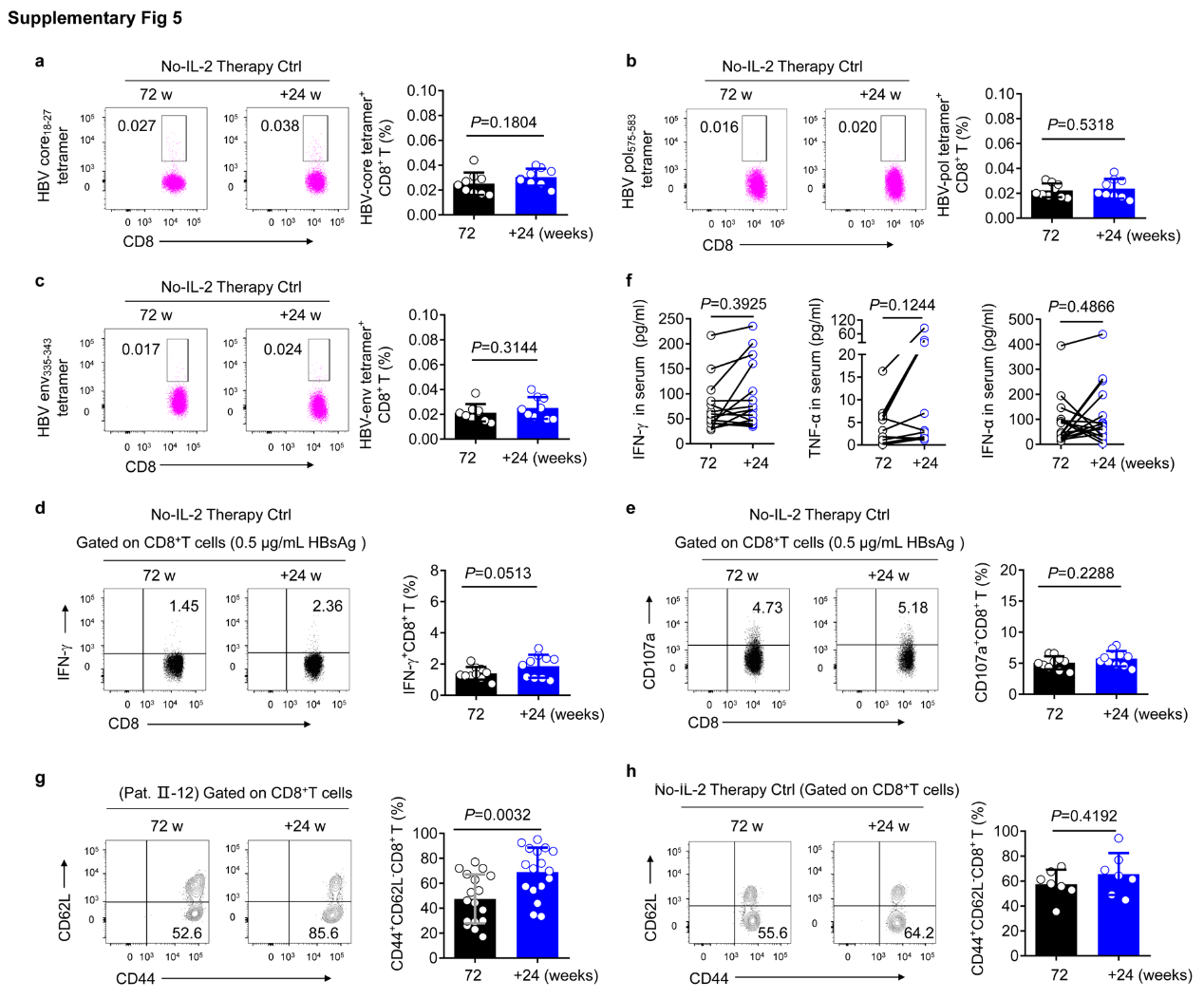


**Supplementary Fig. 5.** **HBV-specific CD8^+^ T cells** **immune responses did not change significantly in patients who did not receive sequential IL-2 therapy. a-c.** Representative density plots (left) and pooled data (right) showing HBV core_18-27_-specific **(a)**, pol_575-583_-specific **(b)** and env_335-343_-specific **(c)** CD8^+^ T cells gated on live CD3^+^ T cells from PBMCs of NR (α-2b) patients at week 72 (72 w; 48 weeks of IFN-α-2b therapy and 24 weeks of follow-up), and week 96 (+24 w; a further 24 weeks without sequential IL-2 therapy); *n* = 9. **d, e.** PBMCs of NR (α-2b) patients at 72 w and a further 24 weeks without sequential IL-2 therapy (+24 w) were treated with HBsAg (0.5 μg/mL) for 3 days. Representative intracellular cytokine staining (left) and pooled data (right) showing IFN-γ **(d)** and CD107a **(e)** expression by CD8^+^ T cells; *n* = 10. **f**. IFN-γ (left), TNF-α (middle), and IFN-α (right) levels in serum were detected by ELISA at 72 w (black), and a further 24 weeks without sequential IL-2 therapy (+24 w) (blue); *n* = 11. **g, h.** Representative density plots (left) and pooled data (right) showing the proportion of CD44^high^CD62L^low^CD8^+^ T cells from PBMCs of NR (α-2b) patients at 72 w and a further 24 weeks with **(g)** or without **(h)** sequential IL-2 therapy (+24 w); *n* = 17 and 10, respectively. For **(a)** to **(h)**, data are analyzed by two-tailed unpaired Student’s t-test; **P* < 0.05; ***P* < 0.01; ****P* < 0.001; *****P* < 0.0001. Data are presented as mean ± SD.


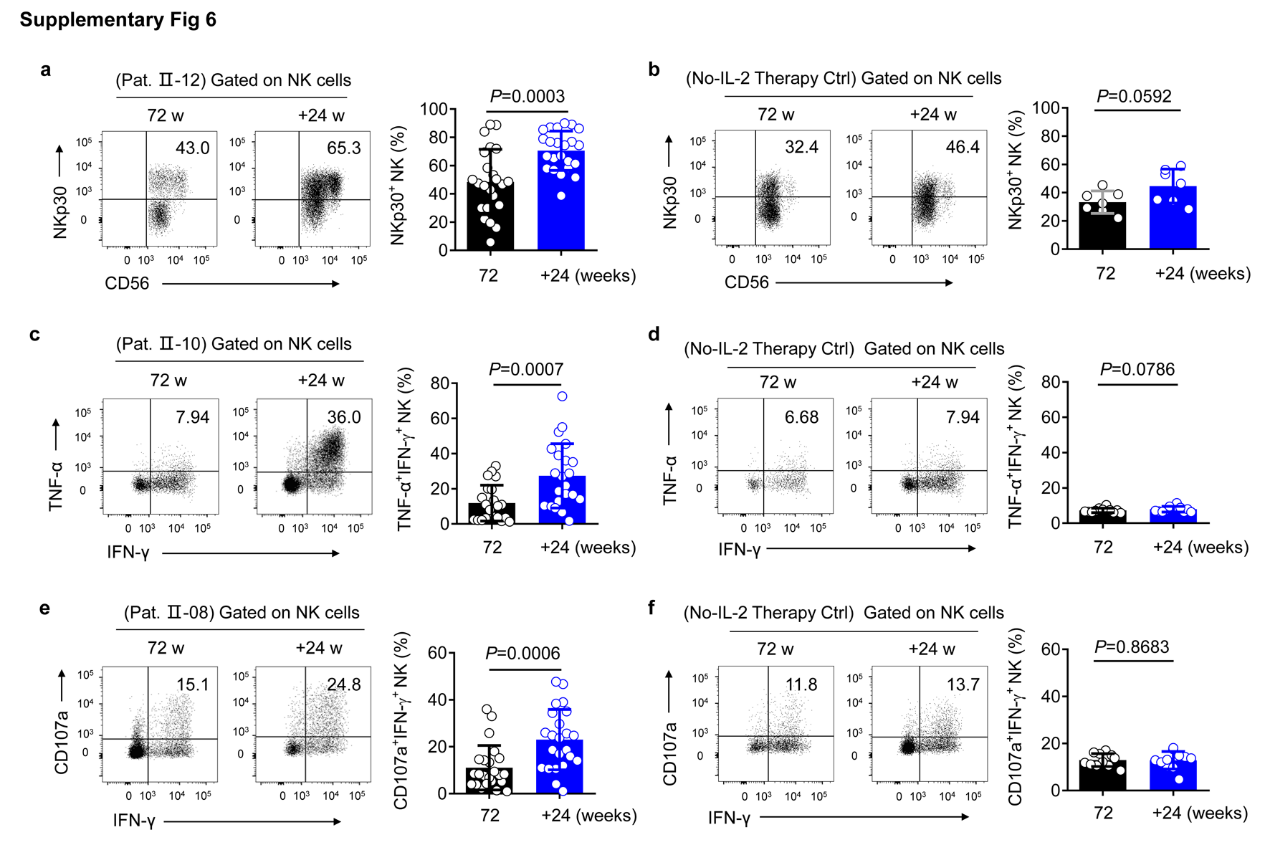


**Supplementary Fig. 6.** **Augmentation of NK cell effector functions after sequential IL-2 therapy *in vivo*.** Data are analyzed and quantified from the PBMCs of NR (α-2b) patients at week 72 (72 w), and a further 24 weeks (+24 w) of sequential IL-2 therapy **(a, c, e)** or No-sequential IL-2 therapy **(b, d, f)**. **a, b.** Representative density plots (left) and pooled data (right) were presented showing the proportion of NKp30 expression by NK cells from the PBMCs of NR (α-2b) patients with **(a)** or without **(b)** sequential IL-2 therapy. **c, d.** Representative intracellular cytokine staining (left) and pooled data (right) were presented showing IFN-γ and TNF-α co-expression by NK cells from the PBMCs of NR (α-2b) patients with **(c)** or without **(d)** sequential IL-2 therapy. **e, f.** Representative intracellular cytokine staining (left) and pooled data (right) were presented showing IFN-γ and CD107a co-expression by NK cells from the PBMCs of NR (α-2b) patients who did **(e)** or did not **(f)** receive sequential IL-2 therapy. For **(c)** to **(f)**, PBMCs were stimulated with phorbol 12-myristate 13-acetate (PMA). For **(a)** to **(f)**, Data are analyzed by two-tailed paired Student’s t-test; **P* < 0.05; ***P* < 0.01; ****P* < 0.001; *****P* < 0.0001. Data are presented as mean ± SD.


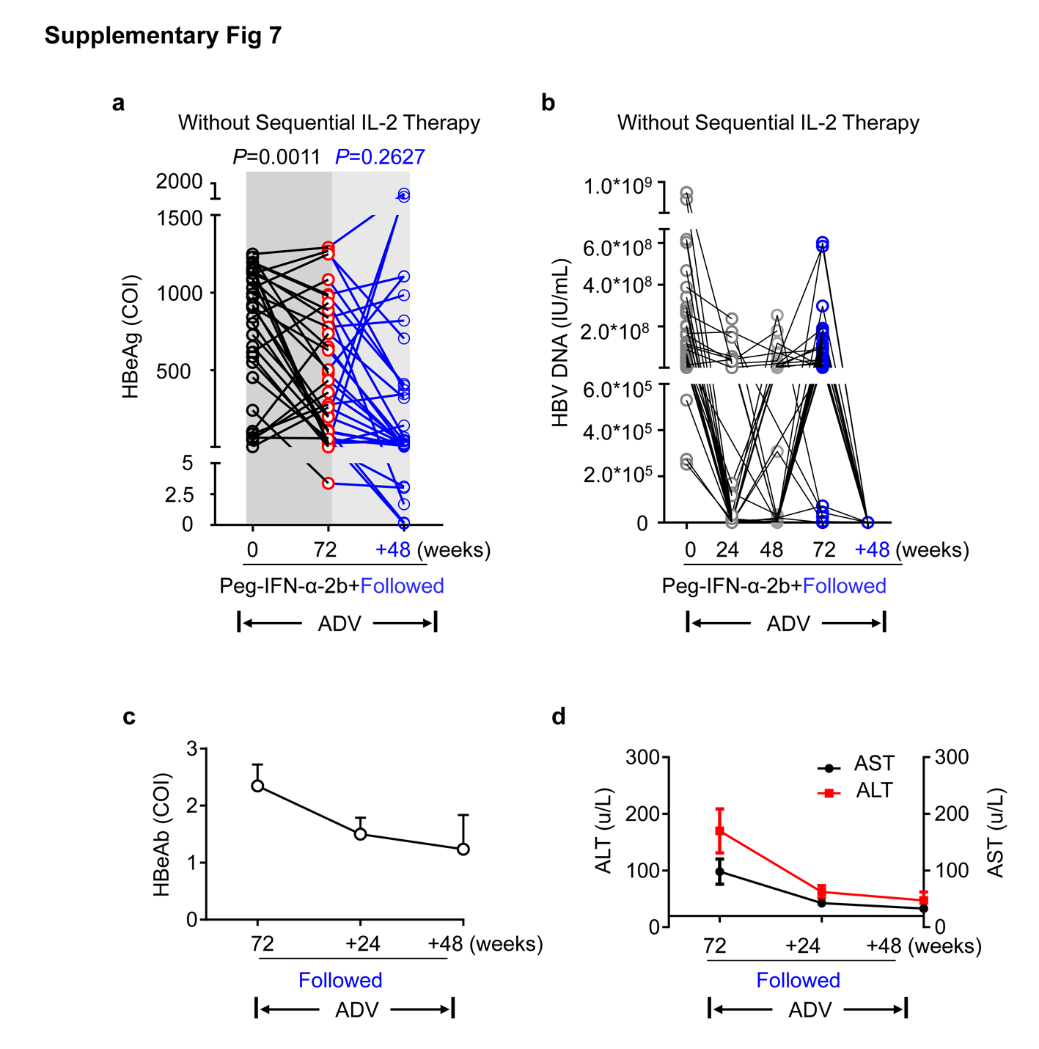


**Supplementary Fig. 7.** **The clinical outcome in NR** **(α-2b) patients without sequential IL-2 therapy *in vivo*. a, b.** Cumulative data showing serum HBeAg levels (COI, cut-off index) **(a)** and HBV DNA levels **(b)** through Peg-IFN-α-2b therapy (48 weeks of Peg-IFN-α-2b + ADV therapy and 24 weeks of follow-up) and follow-up without sequential IL-2 therapy (+48 weeks) in clinical trial 1. **c.** Cumulative longitudinal data for serum HBeAb levels, during IFN-α therapy and follow-up. **d.** The ALT (red) and AST (black) of patients during IFN-α therapy and follow-up. For **(a)** to **(d)**, *n* = 34. Data are analyzed by Wilcoxon matched-pairs signed rank test **(a)**; **P* < 0.05. Data are presented as mean ± SD.
