# Supplementary material for: Restoration of HBV-specific CD8+ T-cell responses by sequential low-dose IL-2 treatment in non-responder patients after IFN-α therapy
Source: Signal Transduct Target Ther. 2021 Nov 5;6:376. doi: 10.1038/s41392-021-00776-0 (PMC8569154; doi:10.1038/s41392-021-00776-0)
Supplement: Supplementary file 2 — Supplementary Table 1 [file 41392_2021_776_MOESM2_ESM.docx]

**Supplementary Table 1. Characteristics of patients treated with Peg-IFN-α-2b in Clinical Trial 1**

| Characteristic |  | Group 1 (n = 45) | Group 2 (n = 47) | *P* value |
| --- | --- | --- | --- | --- |
| Gender (M/F) |  | 37/8 | 29/18 | 0.038 |
| Age (yr) |  | 29 (19–45) | 27 (18–41) | 0.691 |
| HBV Genotype (C/B) |  | 25/20 | 22/24 | 0.531 |
| Median HBV DNA, log IU/mL (range) |  | 7.57 (4–10.2) | 7.30 (4.5–8.9) | 0.740 |
| Median ALT, ULN (range) |  | 204 (84–480) | 231 (80–580) | 0.918 |
| Median HBsAg, log IU/mL (range) |  | 4.00 (2.90–4.70) | 3.97 (1.60–4.90) | 0.942 |
| Median HBeAg, COI (range) |  | 2.60 (0.9–3.1) | 2.31 (0.5–3.1) | 0.439 |
| HBeAg seroconversion (Y/N) |  | 12/33 | 16/31 | 0.501 |

All patients were HBsAg positive, HBeAg positive, Chinese people. Anti-HBeAg seroconversion means HBeAg loss and seroconversion to anti-HBe.

*P* values were calculated by means of the Mann-Whitney U test for continuous variables, and the Chi-Squared and Fisher exact tests for categorical variables.

M, Male; F, Female; COI, cut off index; yr, year; ULN, upper limit of normal; Y, Yes; N, No.

**Supplementary Table 2. Characteristics of patients treated with or without sequential low-dose IL-2 in Clinical Trial 2**

| Characteristic |  | Value |  |
| --- | --- | --- | --- |
|  | Sequential  IL-2 group (n=23) | No-Sequential  IL-2 group (n=15) | *P* value |
| Gender (M/F) | 19/4 | 10/5 | 0.436 |
| Age (yr) | 34.2 (25-49) | 37.6 (24-47) | 0.608 |
| HLA-A2 positive (Y/N) | 13/10 | 9/6 | 0.752 |
| Median ALT before therapy, ULN (Range) | 29.6 (13-54) | 32.4 (19-51) | 0.790 |
| Median HBsAg before therapy, COI (Range) | 2968 (29-8013) | 2573 (46-7859) | 0.984 |
| Median HBeAg before therapy, COI (Range) | 14.1 (3.5-14.8) | 140.0 (7.7-58.3) | 0.0658 |
| Median HBeAg after therapy, COI (Range) | 6.3 (1.3-7.6) | 101.3 (5.4-81.2) | 0.0011 |
| HBeAg seroconversion after therapy (Y/N) | 5/18 | 0/15 | 0.053 |
| HBsAg loss with anti-HBs (Y/N) | 1/22 | 0/15 | 0.413 |

The HBV DNA of the patients in the two groups of Clinical Trial 2 was undetectable.

*P* values were calculated by means of the Mann-Whitney U test for continuous variables, and the Chi-Squared and Fisher exact tests for categorical variables.

NR, non-response; M, Male; F, Female; COI, cut off index; yr, year; HBV, Hepatitis B virus; ULN, upper limit of normal; Y, Yes; N, No.

**Supplementary Table 3. Adverse events during low-dose IL-2 therapy in Clinical Trial 2**

| Adverse events |  | No. of Patients |
| --- | --- | --- |
| Fever in the first week |  | 6 |
| Fatigue |  | 1 |
| Injection-site reaction |  | 8 |
| Infection |  | 0 |

No., number.

**Supplementary Table 4. Antibodies used in flow cytometric analysis.**

| Antibody | Brand | Cat. No. |
| --- | --- | --- |
| PerCP-CY5.5 Mouse Anti-Human CD3 | BD Bioscience | Cat# 340949, RRID:AB_400190 |
| APC-CY7 Mouse Anti-Human CD3 | BD Bioscience | Cat# 557832, RRID:AB_396890 |
| FITC Mouse Anti-Human CD16 | BD Bioscience | Cat# 555406, RRID:AB_395806 |
| PE-CY7 Mouse Anti-Human CD19 | BD Bioscience | Cat# 557835, RRID:AB_396893 |
| FITC Mouse Anti-Human CD24 | BD Bioscience | Cat# 555427, RRID:AB_395821 |
| FITC Mouse Anti-Human CD27 | BD Bioscience | Cat# 555440, RRID:AB_395833 |
| APC-Cy7 Mouse Anti-Human CD14 | BD Bioscience | Cat# 557831 |
| FITC Mouse Anti-Human CD27 | BD Bioscience | Cat# 555440, RRID:AB_395833 |
| APC Mouse Anti-Human CD107a | BD Bioscience | Cat# 560664, RRID:AB_396135 |
| APC Mouse Anti-Human CD137 | BD Bioscience | Cat# 550890, RRID:AB_398477 |
| PerCP-CY5.5 Mouse Anti-Human CD38 | BD Bioscience | Cat# 551400, RRID:AB_394184 |
| FITC Mouse Anti-Human CD4 | BD Pharmingen | Cat# 555346, RRID:AB_395751 |
| FITC Mouse Anti-Human CD8 | BD Pharmingen | Cat# 555366, RRID:AB_395769 |
| PE-Cy7 Mouse Anti-Human CD8 | BD Pharmingen | Cat# 557746, RRID:AB_396852 |
| FITC Mouse Anti-Human IFN-γ | BD Bioscience | Cat# 554700, RRID:AB_395517 |
| PE Mouse Anti-Human CD107a | BD Bioscience | Cat# 555801, RRID:AB_396135 |
| PE Mouse Anti-Human CD69 | BD Bioscience | Cat# 555531, RRID:AB_395916 |
| PE Mouse Anti-Human LT-βR | BD Bioscience | Cat# 551503, RRID:AB_394222 |
| PE Mouse Anti-Human TRAIL | BD Bioscience | Cat# 555531, RRID:AB_393719 |
| PE Mouse Anti-Human PD-1 | BD Bioscience | Cat#560795, |
| PE Mouse Anti-Human Tim-3 | BD Bioscience | Cat# 565570 |
| PE-CY7 Mouse Anti-Human CD56 | BD Bioscience | Cat# 557747, RRID:AB_396853 |
| PE Mouse Anti-Human CD44 | BD Bioscience | Cat# 555479 |
| 7-AAD | BD Bioscience | Cat# 559925 |
| FITC Mouse Anti-Human CD62L | BD Bioscience | Cat# 555543, RRID:AB_395927 |
| APC Mouse Anti-Human HLA-DR | BD Bioscience | Cat# 559866, RRID:AB_398674 |
| PerCP-CY5.5 Mouse Anti-Human CD123 | BD Bioscience | Cat# 558714, RRID:AB_891359 |
| FITC Mouse anti-human Lineage Cocktail | BD Bioscience | Cat# 348801, RRID:AB_10612570 |
| APC-CY7 Mouse Anti-Human CD11c | Biolegend | Cat# 337218 |
| PE Mouse Anti-Human CD132 | BD Bioscience | Cat#555898, RRID:AB_396210 |
| PE Mouse Anti-Human CD25 | BD Bioscience | Cat#555432, RRID:AB_395826 |
| PE Mouse Anti-Human CD122 | BD Bioscience | Cat# 554525, RRID:AB_395453 |
| PE Mouse Anti-Human NKG2D | BD Bioscience | Cat# 557940 |
| PE Mouse Anti-Human TNF-α | BD Bioscience | Cat# 559321, RRID:AB_397219 |
| Alexa Fluor® 647 Mouse Anti-Human CD56 | BD Bioscience | Cat# 557711 |
| Alexa Fluor® 647 Mouse Anti-Human NKP30 | BD Bioscience | Cat# 558408, RRID:AB_398454 |
| PE Phospho-STAT5 (Tyr694) Monoclonal Antibody | eBioscience | Cat# 11-9010-42, RRID:AB_2572520 |
| Alexa Fluor® 488 anti-STAT1 Phospho (Tyr701) Antibody | CST | Cat# 9174  RRID:AB_2198287 |
| FITC Mouse IgG1, κ | BD | Cat# 555748, RRID:AB_396090 |
| PE Mouse IgG1, κ | BD | Cat# 55749, RRID:AB_396091 |
| PerCP-Cy5.5 Mouse IgG1, κ | BD | Cat# 552834, RRID:AB_394484 |
| PE-Cy7 Mouse IgG1, κ | BD | Cat# 557872, RRID:AB_396914 |
| Alexa Fluor 647 Mouse IgG1, κ | BD | Cat# 557714, RRID:AB_396823 |
| APC-Cy7 Mouse IgG1, κ | BD | Cat# 557873, RRID:AB_396915 |
| APC-Cy7 Mouse IgG1, κx | Biolegend | Cat# 400161, RRID:AB_11125373 |
| PE Mouse IgG2a, κ | BD | Cat# 555574, RRID:AB_395953 |
| PerCP-Cy5.5 Mouse IgG2a, κ | BD | Cat# 558020, RRID:AB_396989 |
| PE-Cy7 Mouse IgG2b, κ | Biolegend | Cat# 400325 |

No., number; Cat., catalog; RRID, Research Resource Identifier.
